# The Alkali Tolerance of Broomcorn Millet (*Panicum miliaceum* L.) at the Germination and Seedling Stage: The Case of 296 Broomcorn Millet Genotypes

**DOI:** 10.3389/fpls.2021.711429

**Published:** 2021-08-23

**Authors:** Qian Ma, Caoyang Wu, Shihan Liang, Yuhao Yuan, Chunjuan Liu, Jiajia Liu, Baili Feng

**Affiliations:** College of Agronomy, State Key Laboratory of Crop Stress Biology in Arid Areas, Northwest A&F University, Yangling, China

**Keywords:** alkali tolerance, genotypic variation, phenotype, antioxidant enzymes, soluble osmolytes, cell membrane, broomcorn millet

## Abstract

Broomcorn millet (BM), one of the earliest domesticated cereal crops originating in northern China, can tolerate extreme conditions, such as drought and high temperatures, which are prevalent in saline-alkali, arid, and barren landscapes. However, its adaptive mechanism to alkali stress is yet to be comprehensively understood. In this study, 80 and 40 mM standard alkali stress concentrations were used to, respectively, evaluate the alkali tolerance at the germination and seedling stages of 296 BM genotypes. Principal component analysis (PCA), Pearson's correlation analysis, and *F*-value comprehensive analysis were performed on the germination parameters (germination potential, germination index, germination rate, vigor index, root length/weight, sprout length/weight, and alkali damage rate). Based on their respective *F*-values, the BM genotypes were divided into five categories ranging from highly alkali resistant to alkali sensitive. To study the response of seedlings to alkaline stress, we investigated the phenotypic parameters (plant height, green leaf area, biomass, and root structure) of 111 genotypes from the above five categories. Combining the parameters of alkali tolerance at the germination and seedling stages, these 111 genotypes were further subdivided into three groups with different alkali tolerances. Variations in physiological responses of the different alkali-tolerant genotypes were further investigated for antioxidant enzyme activity, soluble substances, malondialdehyde (MDA) content, electrolyte leakage rate, and leaf structure. Compared with alkali-sensitive genotypes, alkali-tolerant genotypes had high antioxidant enzyme activity and soluble osmolyte content, low MDA content and electrolyte leakage rate, and a more complete stomata structure. Taken together, this study provides a comprehensive and reliable method for evaluating alkali tolerance and will contribute to the improvement and restoration of saline-alkaline soils by BM.

## Introduction

Soil salt-alkalization is a major abiotic stress that restricts global crop production and sustainable agricultural development (Zhu, 2016). Currently, the global salinized land area accounts for 20% of irrigated land, which causes annual agricultural economic losses of up to USD 27.3 billion (Qadir et al., 2014). With the deterioration of the global environment and unreasonable human activities, soil salt-alkalization is becoming increasingly serious. There are three types of soil salinization: Salt stress refers to the stress caused by neutral salt (NaCl and Na_2_SO_4_) in plants (Xu et al., 2019); alkaline stress refers to the stress caused by alkaline salts (NaHCO_3_ and Na_2_CO_3_) in plants (Yang Y. et al., 2019); and saline-alkali stress refers to the stress caused by both neutral salt and alkaline salt in plants (Shi and Sheng, 2005). Na^+^, ${\text{SO}}_{4}^{2-}$, Cl^−^, ${\text{HCO}}_{3}^{-}$, and ${\text{CO}}_{3}^{2-}$ are the major toxic ions to plants in saline soils (Xiao et al., 2020). Generally, salinized soils contain neutral and alkaline salts. Studies have shown that saline-alkali stress may adversely affect plants (whole plants, tissues, cells, sub-cells, molecules, etc.), which seriously affects the yield, quality, and benefits of crops (Xiao et al., 2020; Yuan et al., 2021). Nevertheless, extensively salt-alkalized lands are also important agricultural reserve land resources. Improving and exploiting saline-alkali lands could increase arable land area and relieve the pressure exerted on existing land resources by humans. Therefore, controlling and mitigating the harmful effects of soil salt-alkalization have attracted the attention of researchers and other stakeholders.

In Northeast China, the grassland alkalization rate has exceeded 70% indicating the seriousness of soil alkalization (Yang et al., 2008) and necessitates more attention. However, to date, most research studies have only focused on NaCl and/or Na_2_SO_4_ (Lin et al., 2017; Jia X. et al., 2019; Xiao et al., 2019; Chen et al., 2020; Wang et al., 2020). Asensi-Fabado et al. (2015) reported that salt stress interferes with the normal physiological metabolism of plants, leading to a lack of cellular energy and oxidative stress. Furthermore, Gill and Tuteja (2010) claimed that chemical damage caused by exposure to reactive oxygen species (ROS) eventually leads to plant cell death. To mediate these environmental stressors, Niu et al. (2018) found that elevated oxidase activity is an important physiological factor for plant salt tolerance. Yuan et al. (2021) believed that salt-tolerant broomcorn millet (BM; *Panicum miliaceum* L., 2*n* = 4 = 36) resists salt stress *via* modulation of cell wall biosynthesis and Na^+^ balance. A few other studies have also researched the adaptive mechanism of alkali stress in plant species. These alkali tolerance studies were related to the physiological responses (Jia X. M. et al., 2019), gene expression (Zhang et al., 2013), metabolome, and proteome (Rui et al., 2016; Han et al., 2019). However, no study has investigated the effect of an alkaline buffer system (molar ratio NaHCO_3_:Na_2_CO_3_ = 9:1) on crops of different genotypes. It was generally believed that in addition to osmotic stress and ionic toxicity, plants in alkalized soils will also encounter high pH stress (Xiao et al., 2020), though the latest research study showed that alkali stress on plants is mainly dependent on the specificities of the ${\text{HCO}}_{3}^{-}$ in the buffering system, which complicates the discovery of plant adaptation mechanisms in alkalized soils. This is due to the difficulty in determining from which stress factors the plants have suffered and what key stress factors lead to certain physiological or molecular changes. Therefore, investigation of these physiological responses of plants to alkaline stress may promote the recognition of phytoremediation and bring us closer to the restoration and improvement of alkaline soils.

Phytoremediation is a method of reducing harmful substances in the soil by separating pollutants through absorbing, transferring, extracting, or fixing harmful substances, during plant growth. Phytoremediation is a safe and reliable method for developing low-cost green vegetation with the ability to treat soil pollution, with good economic and ecological benefits (Alaribe and Agamuthu, 2015). Therefore, phytoremediation is a sustainable and well-applied method for soil environment remediation. Currently, various preliminary studies researched tolerant plant candidates for heavy metal and salt-stress repair, which have been met with favorable results (Huang et al., 2019). When plants are subjected to salt-alkali stress, they resist stress and initiate a series of stress responses to maintain their normal physiological metabolism (Xiao et al., 2020). These reactions are manifested as changes in related physiological indicators, which characterize the degree of stress on the cell, the ability to reduce toxicity, and the tolerance to stress. Therefore, cultivating alkali-tolerant varieties plays a pivotal role in the restoration of saline-alkali soils and sustainable development.

Broomcorn millet, one of the earliest domesticated cereal crops originating in northern China (Zhang Y. et al., 2019), can tolerate extreme conditions such as saline-alkali, drought, and high temperature, prevalent in saline-alkali, arid, and barren landscapes (Hunt et al., 2014; Yuan et al., 2021). However, few studies exist on the adaptive mechanism of BM to abiotic stress, and those available focused on fertilizer (Liu et al., 2020), drought (Zhang D. Z. et al., 2019), and neutral salt stress (Liu et al., 2015; Yuan et al., 2021). For instance, Yuan et al. (2021) revealed the effect of 0.1% NaCl stress on the growth of BM. Furthermore, Liu et al. (2015) proved that there is a large genotypic variation in the salt tolerance of BM. However, the alkali-tolerant genotype of BM has not yet been evaluated and identified.

This study evaluated the alkali tolerance of different genotypes of BM to promote restoration and improve productivity in alkaline soil. To this end, 296 BM cultivars were selected, their physiological changes in response to alkaline stress during the germination and seedling stages were studied, and their tolerance was evaluated. In particular, this study determined the following: (a) the optimal evaluation method of alkali tolerance for 296 core BM genotypes through analyzing seed germination traits under mixed alkali conditions; (b) the seedling growth performances of different alkali-tolerant genotypes selected according to their tolerance at the germination stage to explore BM physiological characteristics; and (c) the alkali-tolerant genetic resources from the core genotypes of BM. This study will provide a theoretical basis for the application of BM restoration and improvement of alkaline soils.

## Materials and Methods

### Plant Materials

A total of 296 BM core collections (Supplementary Table 1), including landraces and cultivars, were selected as materials for this study. Of these, 288 varieties came from China, 2 from the United States, 2 from the former Soviet Union, 2 from Poland, and 2 from India. All materials were provided by the College of Agronomy, Northwest A&F University, Shaanxi, China.

### Experimental Design

#### Germination Stage

Seeds with similar sizes and appearances were sterilized with 0.1% HgCl_2_ for 5 min, rinsed five times with sterile water, and sown in Petri dishes with double-layered filter paper after the surface water was absorbed by filter paper. In each Petri dish, 50 seeds were neatly placed and cultivated in either 8 ml distilled water (control) or a mixed alkali with a concentration of 80 mM (molar ratio NaHCO_3_:Na_2_CO_3_ = 9:1). The seeds were germinated by hydroponics culture in a controlled greenhouse incubator (30°C day/18°C night, 14 h light/10 h dark cycle, and 60% relative humidity). The distilled water and mixed alkali were replaced every 24 h.

#### Seedling Stage

Seeds with similar appearance and size were sterilized, washed in a similar fashion as the germination period, and then cultured in a Petri dish (distilled water) with double-layer filter paper for 1 day at 30°C. The germinated seeds were sown on a seedling identification instrument and cultured in 1/2 Hoagland nutrient solution in a greenhouse under controlled conditions (30 ± 1°C day/18 ± 1°C night temperature, 24,000 l × illumination intensity, 14 h light/10 h dark cycle, and 55–60% relative humidity). The seedlings at the three-leaf one-heart stage were transferred to a nutrient solution with 40 mM (molar ratio NaHCO_3_:Na_2_CO_3_ = 9:1) mixed alkali for stress. A 1/2 Hoagland nutrient solution without alkali was used as a control. Plants were harvested from three biological replicates to observe and record the changes between the different treatments after 5 days of stress.

### Measurements of Plant Growth

#### Germination Stage

The plants exposed to the two different treatments were grown for 5 days under the above-mentioned culture conditions. The germination standard is for seeds with a germ length greater than or equal to 1/2 the seed length and seeds with radicle greater than or equal to the seed length. The number of seeds that germinated on the fourth day was counted. On the seventh day, a vernier caliper was used to measure the length from the seed embryo to the longest root tip [the root length (RL)] or shoot tip [sprout length (SL)]. Furthermore, moisture was absorbed on the root or sprout surface, the root fresh weight (RW) and sprout fresh weight (SW) were measured using an electronic balance, and the relative alkali damage rate was calculated. Three replicates for a single parameter and three independent replicates of each treatment were considered to evaluate the different parameters under the same experimental conditions. The relevant calculation formulas are as follows:

$$\begin{array}{l} \text{Germination potential }\left(\text{GP}\right)\\ =\left(\frac{\text{number of germinated seeds on the 4th day}}{\text{number of tested seeds}}\right)\times 100\\ \text{Germination rate }\left(\text{GR}\right)\\ =\left(\frac{\text{number of germinated seeds on the 7th day}}{\text{number of tested seeds}}\right)\times 100\\ \text{Relative alkali damage rate }\left(\text{RAD}\right)=1-\left(\frac{\text{alkali GR}}{\text{control GR}}\right)\times 100\\ \text{Germination index }(\text{GI})=\sum \left(\frac{G_{\text{t}}}{D_{\text{t}}}\right) \end{array}$$

where *G*_*t*_ is the number of germinated seeds on day *t*, and *D*_*t*_ is the corresponding day.

$$\begin{array}{l} \text{Vigor index }\left(\text{VI}\right)=\text{RW}\times \text{GI} \end{array}$$

#### Seedling Stage

Plant height (PH), stem thickness, green leaf area, RL, fresh weight, and root structure were measured on the fifth day after stress. A ruler was used to measure the PH and RL, and a vernier caliper was used to determine stem thickness. After absorbing the surface moisture, RW and SW were measured using an electronic balance. The roots were sampled and rinsed, after which the clean roots were placed on a glass dish filled with water and scanned using an Epson Perfect V700 Pro scanner (Seiko Epson, Suwa, Japan). The total root length (TRL) (cm), root surface area (cm^2^), and root volume (cm^3^) were analyzed using the WinRHIZO 2017 software (Reagent Instruments, Quebec, Canada).

### Measurements of Electrolyte Leakage Rate and Malondialdehyde Content

The estimation of electrolyte leakage at the leaves was performed according to the method described by Nishiyama et al. (2011) with slight modifications. Briefly, fresh leaves were cut into suitable smaller sections and placed in a clean graduated test tube. Double-distilled water (10 mL) was added and left to stand for 2 h at 25°C. Then, the electrolyte leakage EC1 was measured with a conductivity meter (DDS-307A). The test tubes were incubated in boiling water for 10 min, and then, EC2 was determined after cooling to 25°C. The percentage of electrolyte leakage was determined as the percentage of conductivity before and after boiling of the detached roots.

The malondialdehyde (MDA) content in BM leaves was measured according to the method described by Heath and Packer (1968). Fresh samples were homogenized in trichloroacetic acid (5%, w/v) and centrifuged at 11,500 g for 12 min. The mixtures were incubated with 0.5% thiobarbituric acid solution (prepared in 20% trichloroacetic acid) in a ratio of 1:4, incubated in boiling water for 30 min, and then immediately placed on ice to cool. MDA was quantified using an extinction coefficient of 155 mM^−1^ cm^−1^ after reading the absorbance differences at 532 and 600 nm.

### Measurements of Organic Osmolytes (Soluble Sugar, Soluble Protein, and Proline)

To measure proline content in BM leaves, the method described by Yuan et al. (2021) was slightly modified. The homogenate was prepared by taking a fresh seed sample (0.5~1 g) in an aqueous solution of sulfosalicylic acid (3%, w/v), which was centrifuged at 10,000 g for 15 min. The mixed solution (glacial acetic acid:acid-ninhydrin:supernatant = 1:1:1) was incubated at 100°C for 1 h and immediately transferred to an ice bath to terminate the reaction. The colored chromophore was extracted with toluene, and the absorbance was measured at 520 nm. Proline content was calculated using a graph prepared with proline standard products.

The phenol sulfuric acid (C6H5OH/H_2_SO_4_) method described by Dubois et al. (1956) was used to determine the total soluble sugar content in seeds. In short, 10 ml 80% ethanol was used to extract a fresh sample (0.5~1 g). After centrifugation, the supernatant was diluted with distilled water to 50 ml. Then, 0.5 ml extract, 2.5 ml C6H_3_OH solution (V/V = 5%), and 0.5 ml concentrated H_2_SO_4_ were mixed. After heat treatment in boiling water for 20 min, the mixture was cooled to 25°C. The absorbance was measured at 490 nm using an orange–yellow solution. A standard graph with a series of glucose standard solutions was plotted to calculate the total soluble sugar content.

The soluble protein was extracted according to the protocol of Bradford (1976), and potassium phosphate (K/P) buffer (50 mM, pH 7.0), ascorbic acid (AsA, 1 mM), potassium chloride (100 mM), glycerol (10%, v/v), and β-mercaptoethanol (5 mM) were used to extract the homogenate. After the homogenate was centrifuged at 12,000 g for 15 min, the supernatant was collected for measurement of soluble protein content.

### Measurements of Enzyme Activity

To measure the activity of antioxidant enzymes, superoxide dismutase (SOD), peroxidase (POD), and catalase (CAT) were determined as previously described (Mostofa et al., 2020). The first and second fully expanded leaves and terminal sprouts of the plant were collected. Fresh plant tissue (0.5 g) was homogenized in 3 ml Tris buffer (50 mM, pH 7.8) containing 1 mM ethylenediaminetetraacetic acid (EDTA)-Na and 7.5% polyvinylpyrrolidone at 0~4°C. The homogenates were centrifuged at 10,000 *g* for 20 min at 4°C. Absorbance was measured using a spectrophotometer at 25°C to determine the activities of SOD, POD, and CAT.

### Scanning Electron Microscopic Examination of Leaf Structure

To observe the leaf structure, ~1/3rd of the penultimate leaf was taken, washed with deionized water, dehydrated with gradient ethanol of different concentrations, and then dried. The samples were sputtered with gold/palladium at a ratio of 60:40 and observed under a scanning electron microscope (S4800, Hitachi, Tokyo, Japan).

### Data Analysis

In this study, all data were expressed as the mean ± SE of three replicates. Data were analyzed with SPSS (Statistical Product and Service Solution) statistical software version 19.0 (IBM, Chicago, IL, USA), and the least significant difference test was used to determine the difference between the means. All statistical analyses were performed at *P* < 0.05. The effect of variety on variables was analyzed using one-way ANOVA. To accurately evaluate the physiological response of plants to alkali stress, most of the parameters in this study included germination rate (GR), germination index (GI), germination potential (GP), RL, SL, RW, SW, vigor index (VI), and relative alkali damage rate (RAD) expressed as relative values of 80 mM or 40 mM (Na_2_CO_3_:NaHCO_3_ = 9:1) per plant, according to the method of Liu et al. (2020).

The relative values of physiological traits were used for principal component analysis (PCA) and cluster analysis to evaluate the different tolerances of BM genotypes. The F-values were calculated according to the results of PCA, which represent the physiological response of different varieties to alkaline stress. Pearson's correlation coefficient was used to determine the relationship between alkali tolerance and physiological parameters. All figures were drawn using the Origin Pro 2020 (OriginLab, Northampton, MA, USA).

The scoring formula of each principal component can be obtained based on the system matrix of the composition scoring of the PCA.

$$\begin{array}{l} F_{i}=\sum_{i=1}^{n}\left(X_{i}\times S_{i}\right)\;i=1,2,3\ldots \ldots n \end{array}$$

where *F*_*i*_ is the index weight of the *i*th principal component, *X*_*i*_ is the relative value of the *i*th parameter, and *S*_*i*_ is the score of the *i*th parameter in the first principal component.

$$\begin{array}{l} F_{j}=\sum_{j=1}^{n}\left(X_{i}\times S_{j}\right)\;j=1,2,3\ldots \ldots n \end{array}$$

where *F*_*j*_ is the index weight of the *j*th principal component, *X*_*i*_ is the relative value of the *i*th parameter, and S_*j*_ is the score of the *j*th parameter in the second principal component.

$$\begin{array}{l} F_{k}=\sum_{k=1}^{n}\left(X_{i}\times S_{k}\right)\text{k =}1,2,3\ldots \ldots \text{n} \end{array}$$

where *F*_*k*_ is the index weight of the *k*th principal component, *X*_*i*_ is the relative value of the *i*th parameter, and *S*_*k*_ is the score of the *k*th parameter in the third principal component.

The comprehensive score (*F*-value) of each species can be calculated according to the values of *F*_*i*_, *F*_*j*_, and *F*_*k*_:

$$\begin{array}{l} F=W_{i}F_{i}+W_{j}F_{j}+W_{k}F_{k} \end{array}$$

where *F* and *W* are the corresponding principal component index weight and contribution, and *i, j*, and *k* are the *i*th, *j*th, and *k*th principal components, respectively. *F* was used as the comprehensive evaluation value of the alkali resistance of the BM genotypes. The higher the *F*-value, the stronger the resistance to alkali stress of the BM; the lower the *F*-value, the weaker the resistance to alkali stress of the BM (Zhang et al., 2020).

## Results

### Alkali Resistance Comprehensive Evaluation of 296 Broomcorn Millet Genotypes During Germination

#### Analysis of Alkali Tolerance Coefficients Among Traits of the 296 Genotypes

It was observed that each trait of the different BM genotypes showed significant changes under 80 mM alkali stress. To eliminate the inherent biological differences between different varieties, the alkali resistance coefficient (relative value) that reflects alkali resistance was adopted to characterize the alkali resistance of the varieties in this study (Supplementary Table 2). The relative germination potential (GP) and the relative GR varied from 0 to 71.88% and 1.05 to 74.68%, respectively, which indicated that 80 mM alkali stress significantly affected the germination of BM and even killed the seeds of BM. In addition, the range of relative RL and relative RW was 0–72.88% and 0–76.72%, respectively, which reflected the nonnegligible inhibitory effect of 80 mM alkali stress on root growth after germination of BM.

#### Pearson's Correlation Analysis of Alkali Tolerance Coefficients Among Traits of the 296 Genotypes

**Figure 1:**
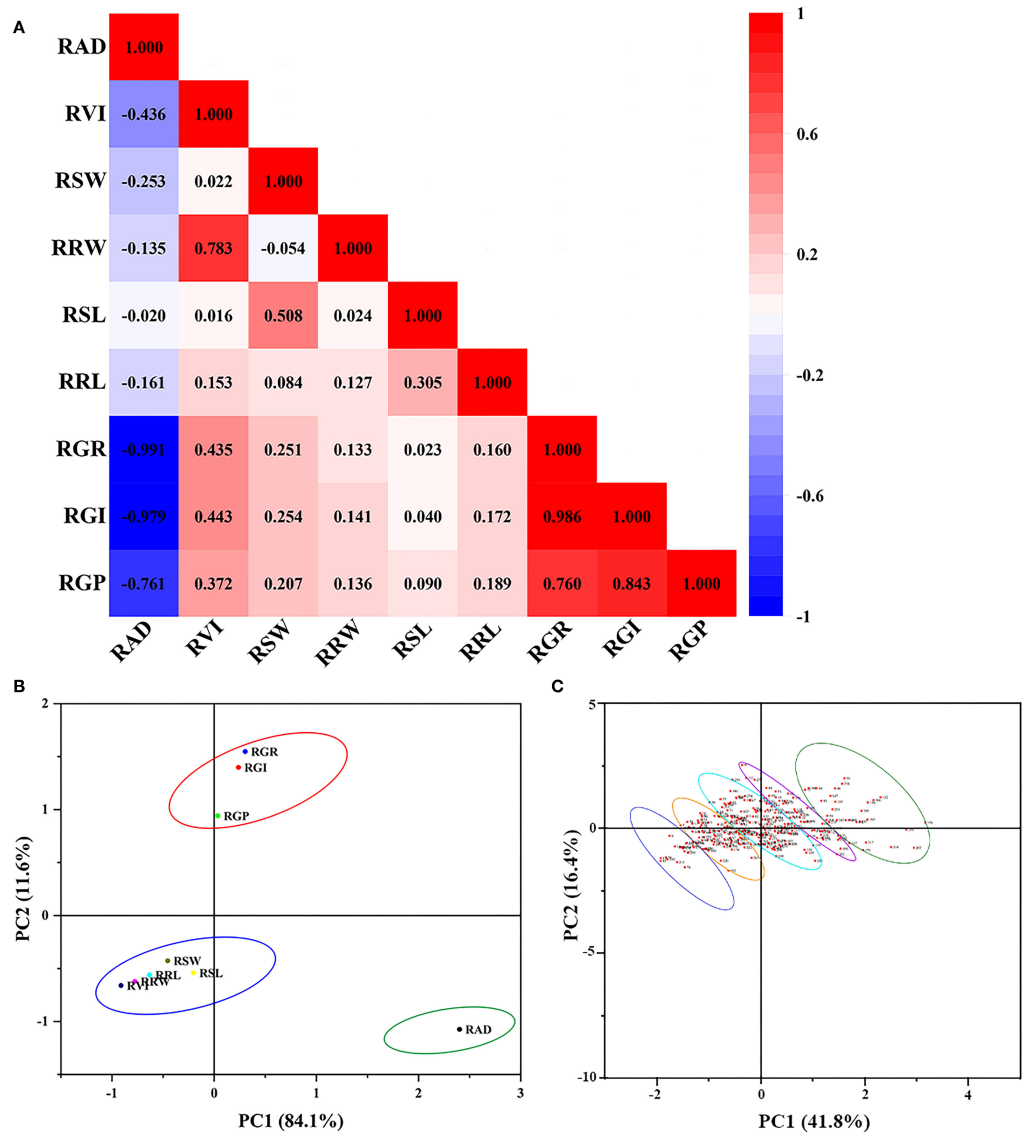
PCC and PCA from 296 genotypes at germination stage. PCC, Pearson's correlation coefficients; PCA, principal component analysis. **(A)** Pearson's correlation coefficients of all traits. **(B)** Principal component analysis of all traits. **(C)** Principal component analysis of 296 genotypes. RAD, relative alkali damage rate; RVI, relative vigor index; RSW, relative bud fresh weight; RRW, relative bud fresh weight; RSL, relative bud length; RRL, relative root length; RGR, relative germination rate; RGI, relative germination index; RGP, relative germination potential.

Pearson's correlation analysis was performed to better understand the characteristics of the alkali tolerance coefficients. Except for the RAD, other biological indicators were positively correlated with each other. The RAD was negatively correlated with other biological indicators, especially the GR (*p* < 0.01, *R* = −0.991). Meanwhile, the GR, GI, and GP were significantly positively correlated with each other (*p* < 0.01), and the correlation coefficients were all above 0.75 (Figure 1A). The evidence indicated that 80 mM alkali stress inhibited the germination of BM seeds to a large extent. However, there may be an overlap of information between the different indicators. Therefore, comprehensive variable indicators will be able to screen alkali-resistant genotypes more effectively.

#### Principal Component Analysis of Alkali Tolerance Coefficients Among Traits of the 296 Genotypes

Dimensionality reduction analysis can eliminate factors that have less impact and greater interference, thereby improving the accuracy of measurement data analysis. Therefore, the above-mentioned single parameters were converted into a more effective index with a reduced number through PCA, based on the alkali resistance coefficient of the 296 BM genotypes. The principal components were extracted according to the principle that the eigenvalue was >1. As shown in Supplementary Table 3, the first principal component, which had the largest contribution rate and eigenvalue, was the relative germination index (RGI) with 45.83 and 4.13%, respectively. Analogously, the second and third principal components with higher eigenvalues were relative fresh root weight and relative SL, with contribution rates of 18.05 and 16.37%, respectively. The cumulative contributions to the total variation of the component from the first to the third principal component reached over 80.25%, which was sufficient to represent a large part of the original indicator information. Therefore, three independent comprehensive indicators can be adapted to objectively analyze the alkali stress tolerance characteristics of the BM.

#### Comprehensive Evaluation of Alkali Tolerance

Comprehensive index scores were obtained according to the PCA results, which were then used for *F*-value analysis. According to the system matrix of composition scoring in Supplementary Table 3 and the formula of each factor mentioned in the “Materials and Methods” section, the total *F*-value was calculated. The alkali resistance of 296 BM genotypes was sorted and clustered according to the *F*-values (Table 1), which were divided into five categories: high alkali resistance, alkali resistance, moderate alkali resistance, alkali sensitivity, and high alkali sensitivity (Figures 1B,C). The order of *F*-values was 1–30, 31–100, 101–200, 201–270, and 271–296, respectively (Supplementary Figure 1A). The *F*-value of the top-ranked varieties was higher, indicating that they have a higher tolerance to alkali stress. In contrast, the *F*-value of the later-ranked varieties was lower, indicating that they had low resistance to alkali stress. To further explore the differences in the alkali tolerance of different BM genotypes, a total of 111 BM genotypes from five categories of alkali-tolerant varieties were selected for seedling alkali-tolerance evaluation, which was the top 30, 30, 20, 20, and 11 varieties of the five categories, respectively.

**Table 1:** The order of alkali tolerance (*F*-value) of 296 genotypes (germination).

**Code**	**Genotype**	**FG**	**Order**	**Code**	**Genotype**	**FG**	**Order**	**Code**	**Genotype**	**FG**	**Order**	**Code**	**Genotype**	**FG**	**Order**
1	Huangmizi	8.235897	83	75	Heihuiruanshu	3.756078	176	149	Qingyangesiniu	7.484232	99	223	Erbaimi	1.391236	216
2	Baimizi	3.803129	174	76	Heiruanshu	−4.91926	286	150	Zhangchuanmamizi	14.7932	18	224	Huangpimi	−4.16322	282
3	Honganchunwei	4.206766	165	77	Hongmizi	4.000995	172	151	Heijizi	14.4233	20	225	A75-2	−0.306	244
4	Anchunwei	−4.96119	287	78	Baishu	−0.90714	255	152	Saigaidesi	6.199388	123	226	B75-5	−2.00586	268
5	Maimizi	0.597981	232	79	Heishu	3.314948	184	153	Huimizi	0.096861	240	227	B75-8	−0.65173	251
6	Xiaomaimizi	5.387373	137	80	Hongruanshu	−3.11121	277	154	Hongyingmi	12.0712	34	228	E75-11	3.195964	189
7	Heimizi	−2.12857	270	81	Bairuanshu	−8.42311	295	155	Huimizi	5.261908	139	229	Jilinshu	−1.77815	266
8	5-Feb	3.085096	190	82	Bendimizi	4.777575	150	156	Hangmizi	0.22093	238	230	Waiyinshu4	7.553773	95
9	Jan-55	0.597302	233	83	Xiaoqingmi	7.527865	97	157	Heimizi	5.00497	144	231	Waiyinshu8	2.591593	194
10	Nenshu23	1.384659	217	84	Huami	2.446268	198	158	Xiaotoumi	9.172057	72	232	A85-6	16.89928	9
11	Shugu	9.96409	61	85	Xiaohuangshu	−1.64443	264	159	Dahuangmi	9.283447	70	233	A85-10	4.438792	159
12	15	−8.53054	296	86	Ziganshu	−0.52901	248	160	Huangmizi	10.0575	59	234	A85-29	3.294865	185
13	6	6.41173	118	87	Shuzi	5.119792	143	161	Heizi	8.823091	78	235	A85-38	4.95669	146
14	2048	−0.19168	242	88	Huangyingshu	2.526804	196	162	Fuyubaimizi	3.402946	183	236	A85-41	7.319341	103
15	2096	−0.30275	243	89	Baimizi	−2.77339	275	163	Hanzhanghuangmizi	10.14975	56	237	A85-45	7.256256	106
16	2275	0.21849	239	90	Huangyingshu	3.510253	180	164	Qiangouhuangmizi	4.983527	145	238	B85-10	10.24233	54
17	2228	−1.31915	261	91	Dangdimi	9.138055	73	165	Heishu	11.67465	38	239	B85-20	4.056054	171
18	Limizi	7.502623	98	92	Huiruanmi	3.480996	181	166	8403/7/2	12.59898	31	240	B85-25	3.266387	187
19	Huangmizi	5.554275	135	93	Ziganhongshu	7.976238	89	167	8311/4/5	0.937772	226	241	B85-68	4.762006	151
20	Heimizi	1.645567	212	94	Yidianhuangshu	−2.16538	271	168	Xiaohongshu	−2.25607	272	242	A75-45	1.856974	209
21	Heimizi	5.145141	142	95	Dahongmi	6.080254	126	169	Xiaohongshu	7.535224	96	243	A75-70	5.640127	132
22	Huangmizi	2.203656	202	96	Bairuanmi	0.899774	228	170	Shuzi	2.548455	195	244	E75-30	−5.23529	291
23	Dahongshu	5.700337	131	97	Heiruanmi	−4.85973	285	171	Mazhayan	−1.57686	263	245	A85-70	−3.11279	278
24	Dabaishu	−0.44643	246	98	Xiaohongruanmi	−0.79697	252	172	Dahuangshu	9.604505	66	246	A85-75	10.62123	49
25	Mazhayan	1.997104	207	99	Huangruanmi	3.254645	188	173	Dazigan	4.722861	153	247	A85-80	12.96612	28
26	Jinxianhuangmizi	0.943488	225	100	Saozhouruanmi	8.992001	75	174	Hejianbaishuzi	5.368157	138	248	A85-88	14.14026	22
27	Huangqimizi	−2.55993	273	101	Hongmi	−5.1129	289	175	Xiaohongshu	10.66675	48	249	A85-101	4.203452	166
28	Humengheinianmi	2.98219	191	102	Huangshuzi	3.418718	182	176	Shuzi	−2.7861	276	250	B85-62	4.756948	152
29	Balinzuogetashu	−1.85749	267	103	Hongmizi	0.035845	241	177	Qinglonghuangshuzi	3.2889	186	251	B85-72	0.261229	237
30	Wuyuanheishuzi	8.191425	84	104	Helanerhuang	2.979337	192	178	Zijibai	−4.82236	284	252	B85-90	3.804888	173
31	Linheshuanglishu	7.91252	90	105	Misuihong	1.854875	210	179	Ukraine shu	8.851814	77	253	Ziganmi	8.275291	82
32	Hanghouxiaoqingshu	9.581102	67	106	Xiaohuangmizi	13.60326	27	180	Huangshu	11.59778	39	254	Yanshu 7	7.622875	93
33	Bamenghuangshuzi	10.56077	51	107	Dahongmizi	4.466021	158	181	Neishuyidianhong	5.742889	130	255	Nianfeng 5	5.77422	129
34	Zhunqijianghuangshu	4.123772	169	108	Xijixiaohuangmi	6.662345	113	182	Taiyuan1036	10.03578	60	256	Nianfeng 7	3.622594	178
35	Yimengliangshu 56-2	8.106347	87	109	Ningmi6	7.019487	108	183	Yu3-39	4.557002	157	257	Yimi 5	7.465593	100
36	Kailubanhuangshu	12.69917	29	110	Zhangyelaohuangmi	7.883519	91	184	Yanpibao	−3.40482	279	258	Yumi 2	15.48533	14
37	Nongwuqingshu 4	9.46106	68	111	Minlehongmizi	7.627278	92	185	Huangmi(shu)	6.614748	115	259	Yumi 3	0.719627	230
38	Fengzhendabaishu	−0.57651	250	112	Jingtaigedahong	−0.81441	253	186	Shuzi	17.38405	7	260	Longshu 21	19.04391	4
39	Helinhongmizi	9.801082	62	113	Yongdengxiaoheimi	−0.43593	245	187	Laoshupishuzi	6.37829	119	261	Longshu 23	−1.2366	260
40	Wuyuanxiaohuangmi	11.52331	41	114	Gaolanbanlianhong	−3.48718	280	188	Laolaihei	6.216976	122	262	Chishu 1	6.184476	124
41	Bamengbaimizi	1.912494	208	115	Jingyuanziganhong	10.58257	50	189	Huangshuzi	1.582723	215	263	Jinshu 1	6.44485	117
42	Bamengheimizi	14.02549	23	116	60-day ziganhongmi	−0.52955	249	190	Nuoshu	−1.48568	262	264	Jinshu 2	0.358581	236
43	Daqidahuangmizi	13.90351	25	117	Huachihuangcaohongmi	13.93627	24	191	Zhadashu	15.14211	15	265	Jinshu 3	11.91954	36
44	Daqiqingmizi	14.41451	21	118	Ningxianzhuyeqinghuangyingmi	7.378873	101	192	Gulangbangehong	−0.48713	247	266	Longshu 10	0.383518	235
45	Zhunqiziganhongmi	4.843033	148	119	Ningxiandahuangnianmizi	0.840343	229	193	Dianxingziganyemi	−1.21907	259	267	Jinshu 4	1.63087	214
46	Yixuandahongmi	15.97619	12	120	Dongxiangduomami	7.329889	102	194	Yemizi	4.302817	162	268	Jinshu 6	7.185081	107
47	Yimengshu75066-5-2	6.6577	114	121	Guanghehuangmi	10.31808	53	195	Haiyuanziganhong	16.45724	10	269	Jinshu 9	9.091928	74
48	Huinonghuangnianshu	3.663517	177	122	Huangmizi	5.606318	133	196	Ningxiahuangmi	21.76194	1	270	Panlonghuangmi	−3.61413	281
49	Gaolanyadanqing	1.120619	223	123	Hongmi	9.618899	65	197	Yangyanjingqingmizi	10.18957	55	271	Ji 2	−1.0749	256
50	Linghehongnianshu	6.845014	109	124	Huangmi	1.29113	221	198	Fengshuang-4	10.4882	52	272	Ji 3	1.058708	224
51	Qingshuinianmizi	9.716388	64	125	Mi	12.63286	30	199	Honghuamizi	5.171773	141	273	Ji 4	4.181443	167
52	Xiaoshuzi	12.43132	32	126	Baimizi	−2.7314	274	200	Shumi(mi)	3.7627	175	274	Longshu 12	6.069781	127
53	Gudoubai	18.22077	6	127	Heimizi	11.55675	40	201	Baishuzi	1.302609	220	275	Longmi 2	9.396219	69
54	Heimizi	8.055509	88	128	Xiaohongmi	7.288866	105	202	Baishuzi	5.20	141	276	Longmi 3	4.40	161
55	Nianmizi	1.736382	211	129	Erhuangmi	8.772988	79	203	Huangmizi	8.98	77	277	Longmi 4	8.18	86
56	Gaoliangshu	−7.33426	293	130	Xiaohongmi	10.81991	45	204	Taiyuan 3164	11.95	36	278	Longmi 7	4.59	156
57	Xiaobaishu	4.124975	168	131	Jinshoushu	4.666374	154	205	Taiyuan 3048	1.63	213	279	71049	10.73	47
58	Liushitianxiaohongshu	2.333107	200	132	Huangjizi	18.93208	5	206	Helandahong	10.13	58	280	Longmi 9	5.41	137
59	Laolaihong	−2.0121	269	133	Baijizi	7.572226	94	207	Gugutoumi	9.18	72	281	Ningmi 8	4.86	148
60	Tiaozaoshu	0.538816	234	134	Xibeitianmizi(shu)	2.043345	206	208	Tulufanmi	8.48	81	282	Ningmi 9	11.52	43
61	Wuzuishu	4.31298	161	135	Heimizi(shu)	14.96699	17	209	Yanbeitianmi	4.09	171	283	Ningmi 10	6.82	112
62	Xiaobaishu	−1.1911	258	136	Xiaobaishu	2.174871	204	210	78	−7.53	294	284	Ningmi 12	2.39	199
63	Dawahui	4.212828	164	137	Xiaohongshu	9.757697	63	211	Zhiduoaosizhi	2.24	201	285	69-422	15.81	14
64	Jiguanshu	11.46037	43	138	Zhengninghongnianmi(shu)	7.291805	104	212	Sechaertuo	3.54	179	286	Ningmi 15	19.79	4
65	Zaoheibai	4.587543	156	139	Bailishu	5.59337	134	213	Huimi	−6.77	292	287	Ningmi 16	20.37	3
66	Huangluoshu	2.118638	205	140	Mazhayan	−0.85202	254	214	790035	4.81	150	288	Ningmi 17	8.46	82
67	Xiaobainianmizi	1.318583	219	141	Baishuzi	6.829345	110	215	790051	11.36	45	289	Liaomi 3	16.96	9
68	Xiaoheishu	−1.17538	257	142	Shuzi	2.188158	203	216	Lahuangmi	8.11	87	290	Liaomi 56	4.22	164
69	Tiaozhouruanshu	6.218708	121	143	Hongruanmi(shu)	6.247378	120	217	Jinmizi	14.54	20	291	Gumi 21	6.70	113
70	Xiaoheishu	6.172835	125	144	Baikemi(shu)	6.491848	116	218	Tuhuangmi	16.34	12	292	Neimi 3	11.91	38
71	Gouweidan	2.955304	193	145	Hongmi(shu)	10.09726	58	219	Niuweihuang	12.264	34	293	Pinmi 1	13.82	27
72	Ruanmizi	1.375843	218	146	034-2	5.835809	128	220	Huimizi	−1.68	265	294	Heitoue	−5.03	288
73	Baishu	2.525024	197	147	Hongmizi	15.12274	16	221	Baigetami	10.69	48	295	4452	−5.14	290
74	Ruanshu	−4.70125	283	148	Langshan 462	1.172671	222	222	Huanglimi	0.93	227	296	Pinmi 2	0.61	231

### Alkali Resistance Comprehensive Evaluation of 111 Broomcorn Millet Genotypes During the Seedling Stage

#### Analysis of Alkali Tolerance Coefficients Among Traits of the 111 Genotypes

Significant changes in each growth parameter of the seedling stage of different BM genotypes under 40 mM alkali stress were observed. Similarly, the relative values were used to characterize the alkali resistance during the seedling period (Supplementary Table 4). The relative value ranges of PH and stem thickness were 40.56–96.56% and 39.8–88.78%, respectively, which proved that 40 mM alkali stress inhibited the growth of the shoots of BM at the seedling stage. In addition, the alkali tolerance coefficients of RL and root volume indicated that 40 mM alkali stress might promote the vertical growth of BM roots in the seedling stage, which were 51.65–142.19% and 0.36–78.36%, respectively. It is noteworthy that the alkali tolerance coefficient of the green leaf area ranged from 0 to 87.97%, and the coefficient of variation was the largest at 81.27%. The results showed that leaf growth was the most sensitive parameter to the 40 mM alkali stress. Therefore, the relative green leaf area can be adapted as the most intuitive phenotypic parameter for evaluating the alkali resistance of BM.

#### Comprehensive Evaluation to Alkali Tolerance of 111 Genotypes During the Germination and Seedling Stages

**Figure 2:**
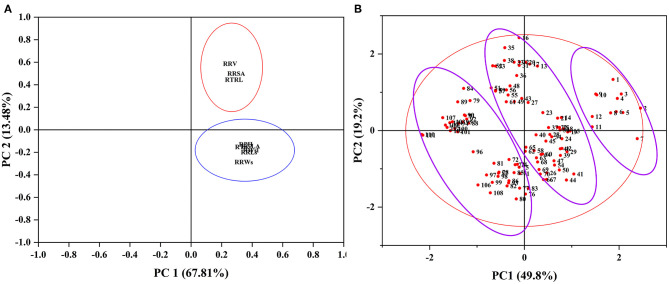
PCA of all traits and alkali tolerance from 111 genotypes at the seedling stage. PCA, principal component analysis. **(A)** Principal component analysis of all traits. **(B)** Principal component analysis of 111 genotypes. RPH, relative plant height; RTS, relative stem thickness; RGLA, relative green leaf area; RRLs, relative root length at the seedling stage; RWsl, relative stem and leaf fresh weight; RRWs, relative root fresh weight at the seedling stage; RTRL, relative total root length; RRSA, relative root surface area; RRV, relative root volume.

**Figure 3:**
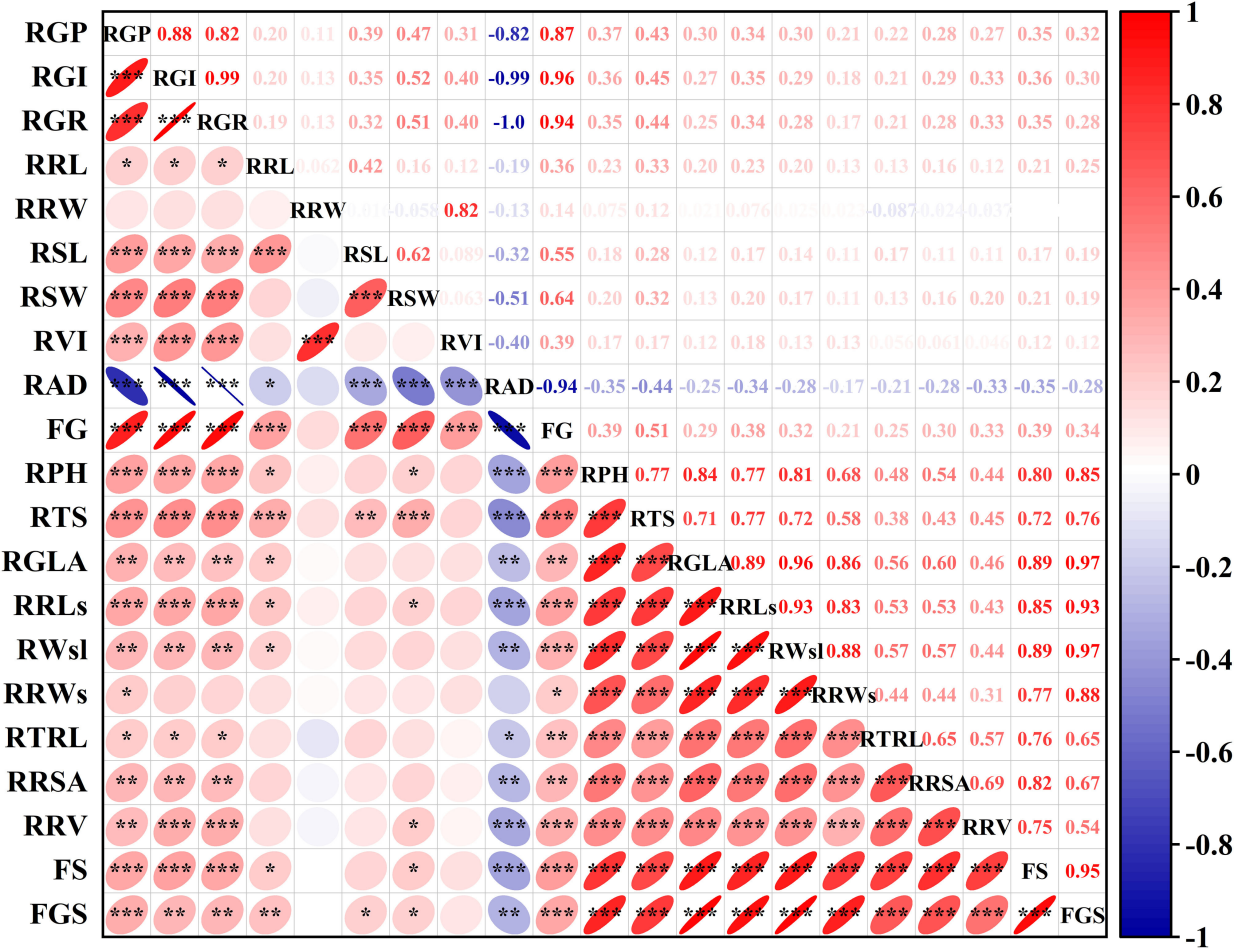
PCA of all traits from 111 genotypes at germination stage and seedling stage. PCA, Principal component analysis. RGP, relative germination potential; RGI, relative germination index; RGR, relative germination rate; RRL, relative root length at germination; RRW, relative root fresh weight at germination; RSL, relative sprout length; RSW, relative fresh sprout weight; RVI, relative vigor index; RAD, relative alkali damage rate; FG, *F*-value at germination stage; RPH, relative plant height; RTS, relative stem thickness; RGLA, relative green leaf area; RRLs, relative root length at seedling; RWsl, relative stem and leaf fresh weight; RRWs, relative root fresh weight; RTRL, relative total root length; RRSA, relative root surface area; RRV, relative root volume; FS, *F*-value at the seedling stage; FGS, comprehensive *F*-value at germination and seedling stage. *P < 0.05; **P < 0.01;***P < 0.001.

To evaluate the alkali tolerance of BM at the seedling stage, PCA was applied to the alkali tolerance coefficient at the seedling stage (Figure 2). The alkali tolerance parameters at the seedling stage were divided into two main components (Figure 2A), with contribution rates of 67.81 and 13.48% (Supplementary Table 6), which represented the growth status of the shoots (stems and leaves) and root traits, respectively. Following the PCA results, the comprehensive evaluation values of alkali tolerance at the seedling stage were calculated (Supplementary Table 7). Furthermore, to comprehensively evaluate the alkali tolerance of BM, the PCA of 111 BM genotypes was carried out by combining the alkali tolerance coefficients at the germination and seedling stages. Four principal components were extracted, with a cumulative contribution of 84.34% (Supplementary Table 5). A total of 111 BM genotypes were subjected to PCA based on the alkali resistance coefficient, which was divided into three categories: alkali-tolerant, moderately alkali-tolerant, and alkali-sensitive (Figure 2B). Pearson's coefficient analysis (Figure 3) of all alkali-tolerance coefficients in the germination and seedling stages revealed that the RAD in the germination stage was negatively correlated with the alkali-tolerance coefficients in the seedling stage. The F-value at the germination stage had a strong positive correlation with both the *F*-value at the seedling stage and the alkali-tolerance coefficients. This showed that the alkali tolerance of the BM at the germination stage positively affected the alkali tolerance at the seedling stage. To further explore the physiological responses of different alkali-tolerant millet varieties, an alkali-tolerant variety (Pm 218), a moderate alkali-tolerant variety (Pm 210), and an alkali-sensitive variety (Pm 213) were selected for physiological analysis. The accuracy of variety selection was supported by cluster analysis at a Euclidean distance of 10 (Supplementary Figure 1B).

### Physiological Responses to Alkaline Stress of Three Broomcorn Millet Genotypes

#### Plant Morphology and Growth

**Figure 4:**
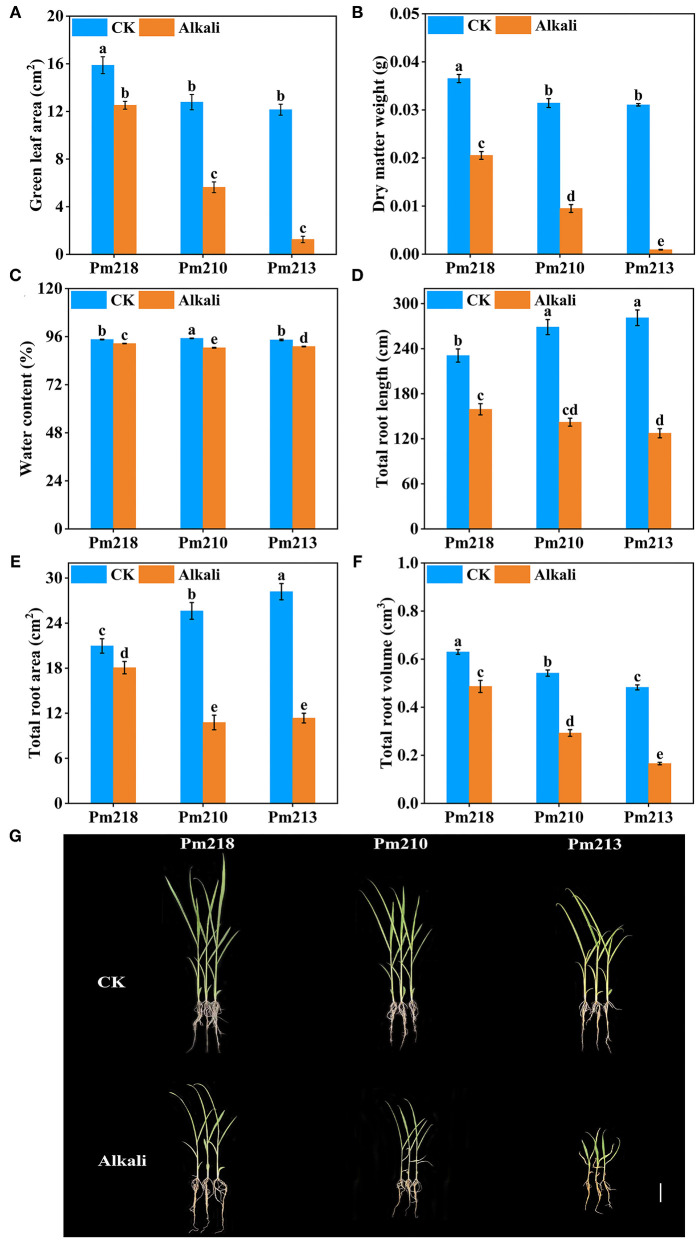
Growth characteristics of three genotypes under control (CK) and alkali stress in the seedling stage. **(A)** green leaf area; **(B)** dry matter weight; **(C)** water content; **(D)** total root length; **(E)** total root surface area; **(F)** total root volume. **(G)** Growth status of alkali-tolerant variety (Pm 218), moderate alkali-tolerant variety (Pm 210), and alkali-sensitive variety (Pm 213) under control (CK) and alkali stress. Bar = 5 cm. Different letters indicate significant differences (*P* < 0.05).

Alkaline stress for 5 days attenuated the plant growth of the three BM genotypes, and the degree of inhibition was different for different varieties. Obvious symptoms of stress were observed on leaf growth, including chlorosis, yellowing, and even death (Figure 4). Figure 4A shows the green leaf area of the three BM genotypes under control and alkali stress. Pm 218 maintains a large green leaf area after 5 days of stress, which indicates that it has a strong ability to adapt to alkali stress. The total dry matter weight and water content of a single plant were significantly reduced by alkali stress (Figures 4B,C). Root scanning analysis results showed that the total RL, root surface area, and root volume of BMs were all inhibited to varying degrees under alkali stress (Figures 4D–F). This demonstrated that, as the first part to be exposed to the alkaline environment, the roots of BMs showed strong sensitivity.

#### Response of Antioxidant Enzyme Activities in Leaves of Different Genotypes to Alkali Stress

**Figure 5:**
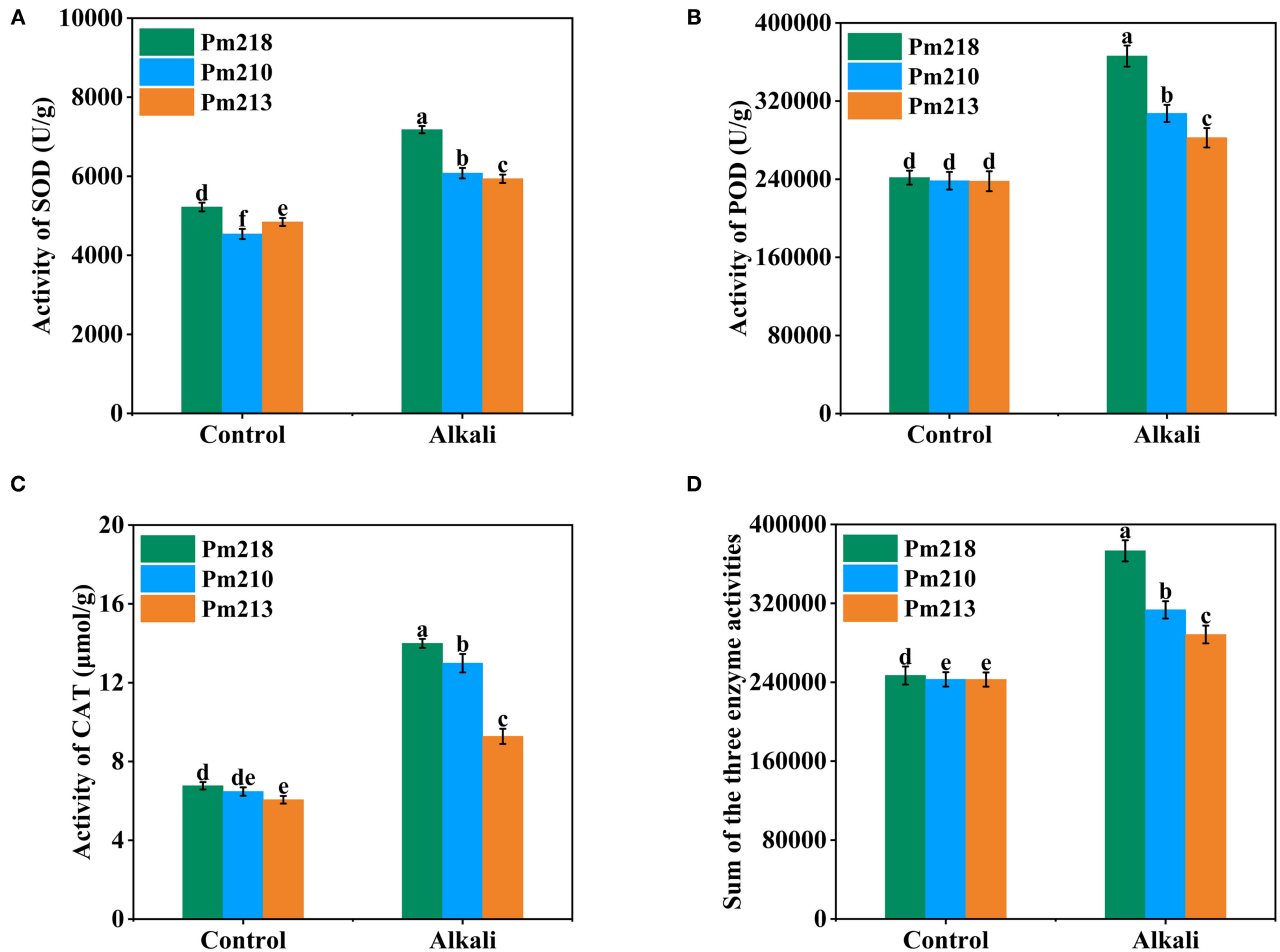
Enzyme activities of three genotypes under control (CK) and alkali stress in seedling leaves. **(A)** Superoxide dismutase activity. **(B)** Peroxidase activity. **(C)** Catalase activity. **(D)** Sum of the three enzyme activities. Different letters indicate significant differences (*P* < 0.05).

To explore the response of BM seedlings to oxidative stress after alkali application, SOD, POD, and CAT were measured (Figure 5). The antioxidant enzyme activity of the three BM genotypes was affected differently by the application of exogenous alkali. In comparison with the control treatment, the activity of SOD in the leaves was significantly increased by the application of alkali (Figure 5A). The same trend was also observed for POD and CAT activity (Figures 5B,C). The activity of CAT in Pm 218 increased through alkali treatment with a maximum elevation of 2.07-fold observed compared with the control. The activity of POD in Pm 213 increased through alkali treatment with a minimum of 1.19-fold observed compared with the control. Furthermore, we performed an additive analysis of the absolute values of SOD, POD, and CAT activity (Figure 5D). Among the three varieties, the alkali-tolerant variety Pm 218 had the highest increase in enzyme activity (1.52-fold) after treatment compared to the control. As expected, the alkali-tolerant variety Pm 213 had the least increase in enzyme activity.

#### Response of Intracellular Compatible Substances in Leaves of Different Genotypes to Alkali Stress

**Figure 6:**
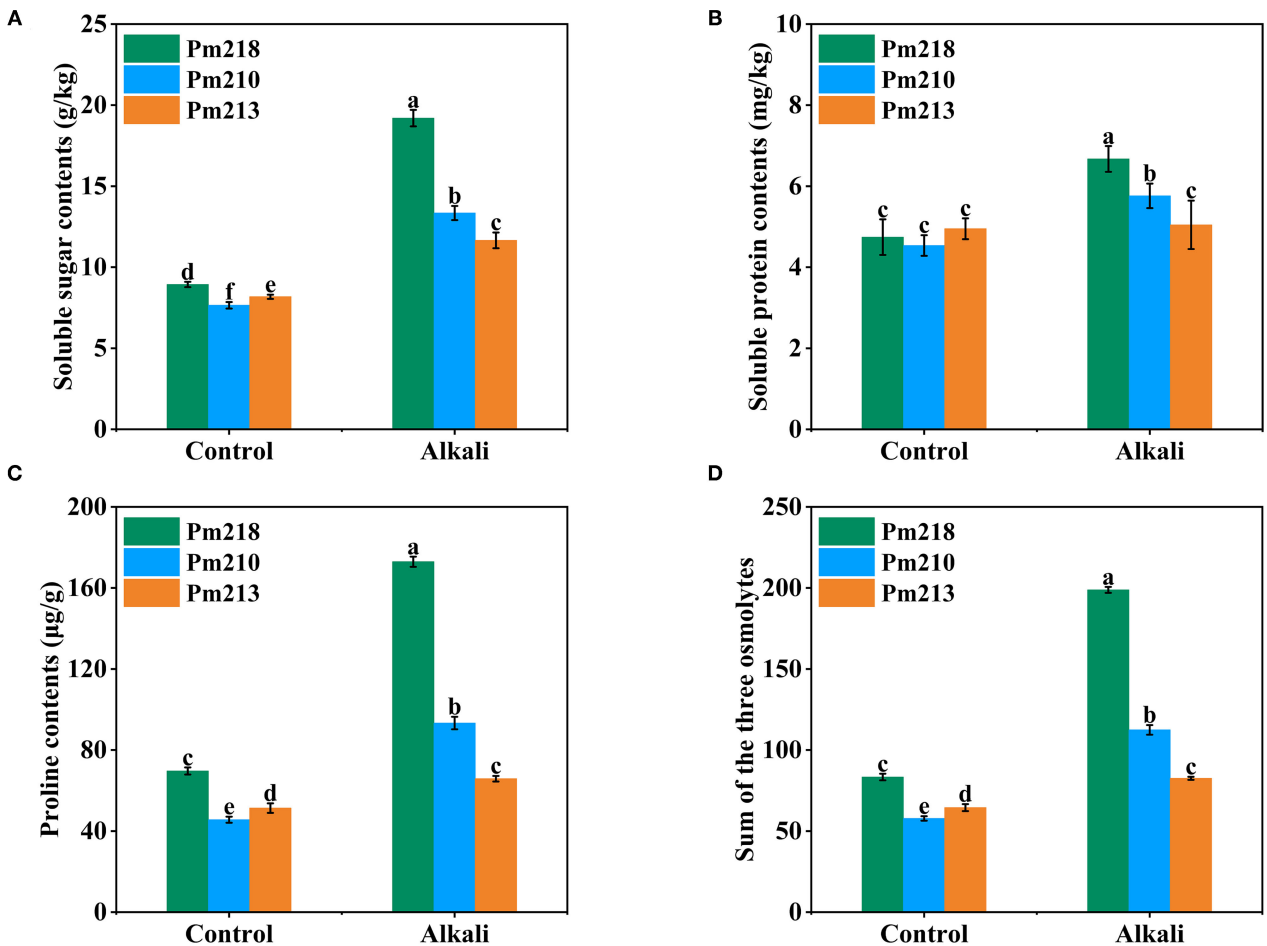
Soluble osmolytes of three genotypes under control (CK) and alkali stress in seedling leaves. **(A)** Soluble sugar contents. **(B)** Soluble protein contents. **(C)** Proline contents. **(D)** Sum of the three osmolytes. Different letters indicate significant differences (*P* < 0.05).

To clarify the effect of alkali treatment on the organic osmotic adjustment substances of BM, the total soluble sugar (Figure 6A), total soluble protein (Figure 6B), and proline content (Figure 6C) were measured after 5 days of exogenous alkali treatment. These contents increased after alkali treatment, especially total soluble sugar and proline. Notably, the application of alkali had no significant effect on the soluble protein of Pm 213. Among the three varieties, the content of total soluble sugar, total soluble protein, and proline increased the most with Pm 218. In addition, we also compared the total absolute values (Figure 6D) of the three osmolytes before and after alkali treatment of the three varieties. By comparison, it was found that after the application of exogenous alkali, the content of osmolytes in the cells of the BM leaves increased drastically, particularly, the alkali-tolerant Pm 218. These results are consistent with the classification of 111 BM genotypes during seedling and germination.

#### Response of Electrolyte Leakage Rate and Malondialdehyde in Leaves of Different Genotypes to Alkali Stress

**Figure 7:**
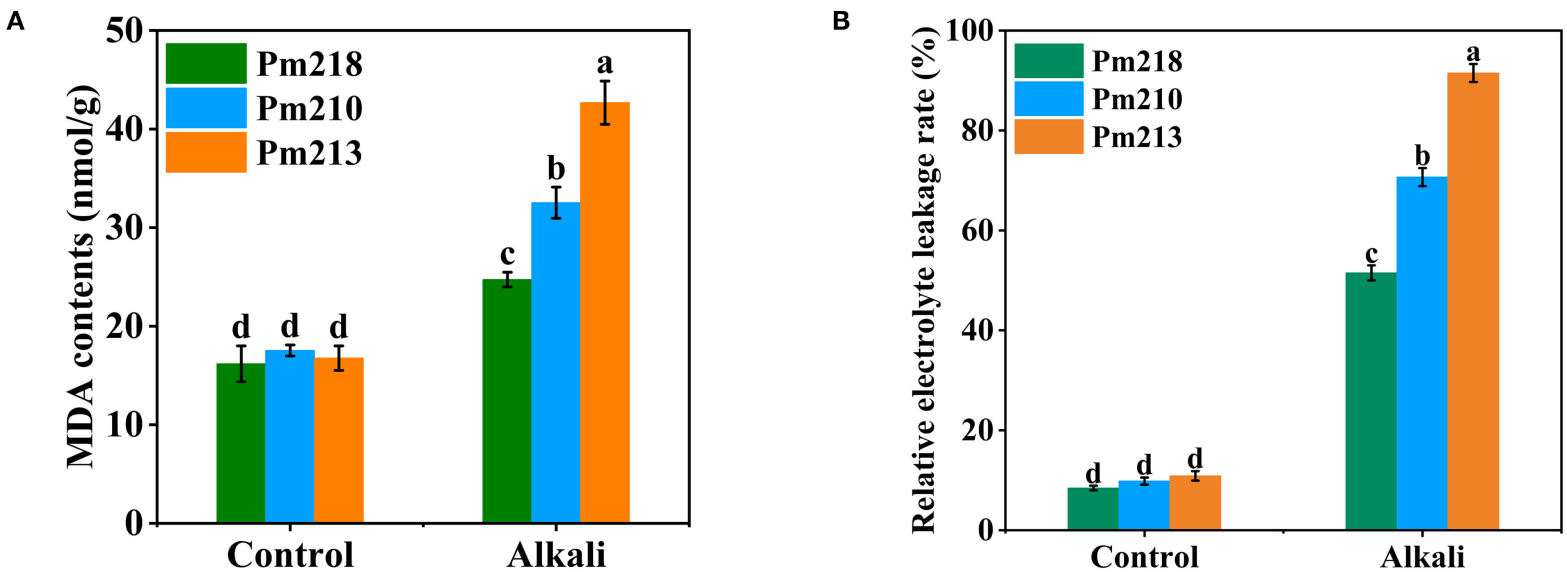
MDA contents and RELR of three genotypes s under control (CK) and alkali stress at seedling. RELR, relative electrolyte leakage rates. **(A)** MDA content. **(B)** Relative electrolyte leakage rate. Different letters indicate significant differences (*P* < 0.05).

To detect the effect of exogenous alkali on cell membrane lipid peroxidation, we tested the MDA content in the leaves of BM seedlings (Figure 7A). The MDA content of seedling leaves was significantly affected by alkali stress, and all three genotypes increased significantly after 5 days of alkali application. Among them, the most affected was Pm 213, followed by Pm 210, and finally Pm 218. This indicated that compared with Pm 213 and Pm 210, Pm 218 maintained a relatively stable cell membrane structure after the administration of exogenous alkali. In addition, we also detected the change in the electrolyte leakage rate of the leaves before and after the alkali treatment (Figure 7B), which coincided with the change in MDA content. These results suggest that exogenous alkali application destroyed the cell membrane structure of the leaves of the seedlings and had an inhibitory effect on the growth and development of seedlings.

#### Responses of Leaf Surface Characteristics of Different Alkali-Tolerant Broomcorn Millet Genotypes to Alkali Stress

**Figure 8:**
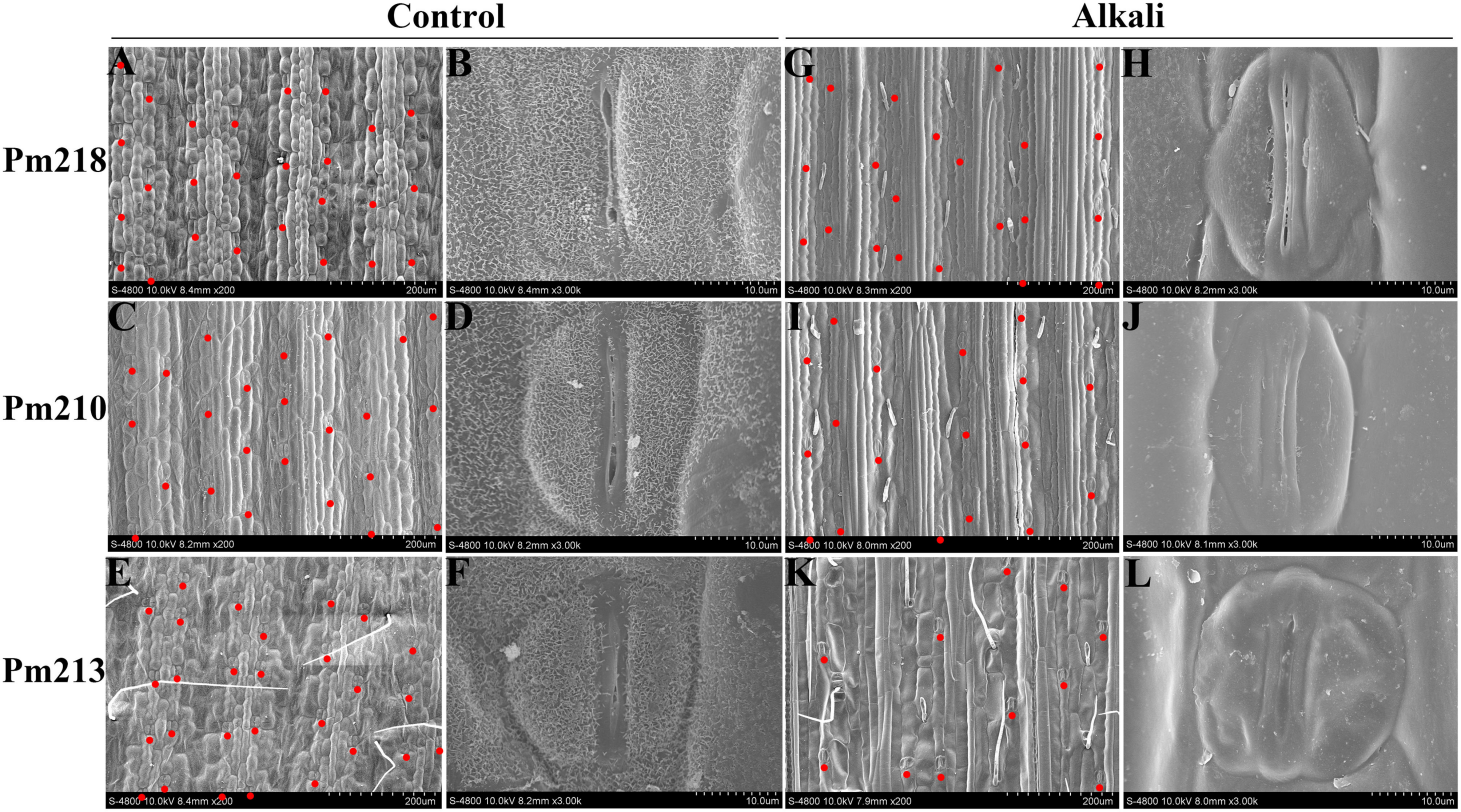
The scanning electron microscope of the leaf surface {**(A–F)**: control condition [**(A,B)**: Pm 218; **(C,D)**: Pm 210; **(E,F)**: Pm 213]} {**(G–L)**: alkali stress [**(G,H)**: Pm 218; **(I,J)**: Pm 210; **(K,L)**: Pm 213]}. The red dot represents the location of the stomata.

Scanning electron microscopy results (Figure 8) showed that stomatal closure occurred in the leaves of BM after the application of exogenous alkali, which helped BM reduce transpiration loss and ion concentration, thus adapting to osmotic stress and ion toxicity caused by alkali stress. Under control conditions, the epidermis of the BM leaf was composed of plump cells arranged along the direction of the leaf veins. These cells formed obvious grooves and ridges on the leaf surface with neatly arranged stomata belts on both sides of the ridges. The stomates were composed of two kidney-shaped accessory guard cells and two dumbbell-shaped guard cells, which were plump, oval, and slightly open (Figures 8A–F). On the surface of the leaves of BM after alkali-stress treatment, neatly arranged but shriveled cells were observed. After alkali stress, the stomatal density decreased, whereas the alkali-sensitive genotype Pm 213 showed the largest decrease. The stomatal guard cells of the highly alkali-tolerant genotype Pm 218 maintained a good state, while the accessory guard cells became deflated (Figure 8H). The stomates were still slightly open to maintain basic physiological functions. In the moderately alkali-tolerant genotype Pm 210, the guard cells and accessory guard cells were deflated and the stomates were tightly closed (Figure 8J). In the alkali-sensitive genotype Pm 213, stomatal guard cells were severely emptied, and accessory guard cells were damaged even after apoptosis (Figure 8L).

## Discussions

### Evaluation of Alkali Tolerance in Broomcorn Millet at Germination and Seedling Stages

In the field of plant abiotic stress, BM is regarded as a pioneer crop because of its drought-tolerant, barren-tolerant, and salt-tolerant properties. Although research studies on the neutral salt tolerance and water stress tolerance of the BM have been reported one after the other, systematic research studies on the alkaline tolerance of the BM have not been reported yet. Furthermore, the response and effective evaluation of different genotypes of BM genotypes to alkali stress at different growth stages are still lacking. Therefore, to rationally utilize and develop the potential of BM as a pioneer crop, it is extremely important to conduct a systematic and comprehensive evaluation of its alkali resistance.

Generally, the response of crops to environmental stress is mainly manifested in two aspects: morphological and physiological characteristics. Seed germination is the primary and sensitive developmental stage of crop growth. Many studies have shown that most plants germinate best under salt-free conditions, as salt stress has an adverse effect on seed germination parameters (Nasri et al., 2011; Hannachi and Labeke, 2018). Therefore, it is particularly important to evaluate alkali tolerance during germination. Yang et al. (2008) believed that salt stress is very different from alkalinity stress. Liu et al. (2015) used GP, RL, SL, relative germination percentage after recovery, relative shoot length after recovery, and relative RL after recovery as identification indicators to evaluate the salt tolerance of 195 BM germination during the germination period and confirmed that salt stress reduced the GR of all BM and inhibited the growth of roots and sprouts during the germination period. Similar results were also observed in this study, that is, alkali stress inhibited germination and suppressed shoot and root growth postgermination. Among the 296 samples of BM studied in this experiment, 100 alkali-tolerant varieties were classified as alkali tolerant, including 30 highly alkali-tolerant and 70 alkali-tolerant varieties, accounting for 23.6% of all research objects. This result will contribute to the research on the alkali resistance of BM.

The seedling stage is also a period in which many crops are very sensitive to stress. Therefore, the growth parameters of seedlings need to be incorporated into the evaluation of alkali resistance. The ability of seedling growth in an alkaline environment is also an important consideration for evaluating alkali resistance. Liu et al. (2015) reported that the survival rate at the seedling stage was negatively correlated with the salt damage rate. In this study, BM genotypes of different genotypes showed similar changes under alkaline stress, but the range of changes was different. Through Pearson's correlation analysis, PCA, and cluster analysis, the alkali tolerance parameters of BM were divided into three factors. The maximum eigenvector load was calculated as the GI, GP, and GR. A total of 111 BM genotypes were selected for comprehensive evaluation of alkali resistance at the seedling stage, which were divided into three categories according to alkali resistance. This diversity in alkali resistance shows its complexity is synergistically derived by genetic and environmental factors. Research studies on salt stress and low nitrogen stress also illustrated the complexity of adversity resistance (Liu et al., 2015, 2020). Therefore, many indicators should be considered when evaluating the alkali resistance of BM. The dimensionality reduction, simplification, and visualization of multidimensional complex traits should be carried out to reflect the alkali tolerance of the BM genotypes in more detail in future research.

Physiological changes are the inevitable result of crops being subjected to environmental stress (Mostofa et al., 2020; Patel and Parida, 2020; Zhao et al., 2020). Changes in physiological indicators, such as the growth and development of BM, can be used to explore the effect of alkali resistance. As expected, after alkali stress, different growth responses of different BM resources were observed. Therefore, according to the alkaline tolerance classification of BM, one variety of each of the three categories was selected to explore the physiological mechanism of BM under alkaline stress.

### Physiological Response to Alkali Application in BM Seedling

Plants exposed to environmental stress have excessive intracellular ROS accumulation, which destroy the organelles and cell membrane structure and cause plant cell metabolism disorders, severely restricting plant growth and development (Grene et al., 2002; Xiao et al., 2020). To resist and adapt to the oxidative damage caused by ROS, plants have developed a set of antioxidant defense systems that are suitable for growth and development (Yang and Yan, 2018). The production of MDA is an indicator of the degree of lipid peroxidation, which reflects the destruction of cell membranes by ROS (Zou et al., 2012). In this study, after 5 days of alkaline stress, the MDA levels in the leaves of all three BM genotypes increased, indicating that the application of exogenous alkali damaged the cell membrane structure of the BM leaves while the BMs were in a state of oxidative stress. The relative electrolyte leakage rate has been used as an indicator of the degree of cell membrane damage under adverse environmental conditions (Abouelsaad et al., 2016; Gao et al., 2020). We observed that the electrolyte leakage rate of BM leaves under alkali stress increased significantly, indicating that alkalinity destroyed the cell membrane structure of the leaves. This resulted in a negative impact on the selective permeability of the cell membrane, thus breaking the original substance exchange state between the internal and external environment of leaf cells. In comparison with Pm 218 and Pm 210, the alkali-sensitive variety Pm 213 had the highest MDA content and relative electrolyte leakage rate after alkali stress for 5 days, implying that its cell membrane structure was damaged the most. This may also be one of the reasons why alkali stress has a greater inhibitory effect on the growth and development of sensitive varieties.

Osmotic pressure imbalance is a result of impaired plant cell membrane function. For proper osmotic pressure, normal water absorption capacity must be maintained, and to avoid physiological drought, plants need to effectively synthesize osmotic regulators in cells (Mostofa et al., 2020; Zhao et al., 2020). The high pH under alkali stress conditions severely inhibits the absorption of K^+^ but promotes the accumulation of Na^+^ in plants. Once the vacuole storage threshold in leaf tissue is reached, the Na^+^ will continue to enter the cytoplasm, which leads to the destruction of biomacromolecules and organelles (Yang Z. et al., 2019). During alkaline stress, higher Na^+^ concentrations were observed in hexaploid wheat leaves, which alleviate the damage caused by alkaline stress in biomacromolecules and organelles through the accumulation of amino acids, carbohydrates, and dehydrin proteins for the maintenance of normal metabolism (Xiao et al., 2020). In this study, the intracellular compatible substance content of leaves, including soluble sugar, soluble protein, and proline, increased after 5 days of exogenous alkali treatment. This result suggests that the leaves of BM can resist and adapt to alkali stress by activating the osmotic regulation system. This is in line with the conclusion that overexpression of *MdTyDc* promotes the accumulation of proline in apples, thereby reducing the damage associated with alkali stress (Liu et al., 2021). Similar conclusions have also been drawn when plants encounter heavy metal stress (Huang and Wang, 2010; Huang et al., 2019).

The antioxidant defense system is composed of antioxidant enzymes that are developed by plants to adapt to various environmental stresses, such as drought stress, salt stress, low-temperature stress, and heavy metal stress (Jayakumar et al., 2014; Czarnockaa and Karpińskia, 2018). Alkaline stress promoted the capacity of plants to scavenge ROS (Jia X. et al., 2019). Under alkaline stress, the expression of calcineurin B-like protein-interacting protein kinase GmPKS4, which regulates the antioxidant system of plants to scavenge excess ROS, was observed in soybeans (Ketehouli et al., 2021). SOD, POD, and CAT are antioxidant enzymes that synergistically scavenge ROS produced by crops under adverse stress to maintain normal physiological and biochemical states (Moez et al., 2016). They play a pivotal role in plant resistance and adaptation to environmental stress. In our study, compared with the control, SOD, POD, and CAT activities increased in the leaves of BM treated with alkali, indicating that alkali stress induces antioxidant enzyme activity to eliminate the accumulation of excessive toxic ROS in the cells. SOD is an important enzyme that decomposes superoxide free radicals into H_2_O_2_ and O_2_ (Yang and Yan, 2018; Mostofa et al., 2020). As higher SOD enzyme activity was also observed in the leaves of BM after alkaline stress, it can also be implied that alkaline stress upregulated the superoxide free radical content in leaves. Similar results have been reported for arsenic stress (Patel and Parida, 2020). POD can catalyze H_2_O_2_-dependent substrates, and CAT catalyzes H_2_O_2_ to generate H_2_O, thereby reducing the toxicity of ROS in plants (Ekmekçi et al., 2008; Huang et al., 2019). We observed that both POD and CAT showed the same trend as SOD activity, which proved that SOD, POD, and CAT actively participate in the elimination of toxic ROS produced by alkali stress. Accordingly, the accumulation of superoxide free radicals and hydrogen peroxide in plants was effectively prevented, to achieve the purpose of reducing the toxicity of ROS to plant cells. In the present study, CAT had the highest rate of change (Figure 5), indicating that CAT plays a major role in regulating and eliminating ROS. Studies have reported that CAT has a stronger regulatory effect than SOD and POD in the presence of high concentrations of heavy metals (Huang et al., 2019). Our results showed that alkaline stress induced the oxidative stress response in BM, which is consistent with the results of previous studies.

Genotypes with different alkali-tolerant abilities showed different antioxidant regulation abilities under alkaline stress. We believe that alkali-tolerant genotypes maintained higher osmolyte synthesis ability and enzyme activity after alkali stress due to more complete cell membrane function and structure, which is consistent with the aforementioned MDA content and relative electrolyte leakage rate. The stable membrane structure maintained normal plant physiological functions, such as protein synthesis, which also contributed to the stability of the antioxidant defense system. The antioxidant enzymes, in turn, respond positively to the elimination of ROS to maintain a normal membrane structure, which promotes alkali tolerance. In addition, we observed that alkali stress reduced stomatal density in the leaves of the three BM genotypes. The alkali-tolerant genotype maintained a good stomata structure with slightly open stomata, while the stomata structure of alkali-sensitive varieties was damaged and closed, which indicated that alkali stress had a destructive effect on the stomata structure of alkali-sensitive BM leaves. The closure of stomata under alkali stress can reduce transpiration loss and maintain proper water potential in cells. Similar results have been reported under salt stress (Albaladejo et al., 2016). In addition, researchers believe that one result of stomatal closure under abiotic stress is an increase in cytoplasmic Ca^2+^ concentration (MacRobbie, 2006), which contributes to the mitigation of osmotic stress and ion toxicity of plants, thereby promoting the ability of plants to resist and adapt to abiotic stress. We believe that this result is consistent with the antioxidant enzyme activity, MDA, and the relative electrolyte leakage rate. Strong antioxidant enzyme activity maintained the synthesis of various enzymes in the cell and the integrity of the cell function and structure, which was conducive to the adaptability and tolerance of plants to alkali stress. Taken together, we suggest that when evaluating the alkali tolerance of different genotypes of BM genotypes, both growth and physiological parameters should be considered. Additionally, the cultivation of BM in saline-alkali land should consider the alkali tolerance of different BM genotypes.

## Conclusions and Limitations

We comprehensively evaluated and categorized the tolerance of 296 BM genotypes to exogenous alkali application by Pearson's correlation analysis, PCA, and cluster analysis. The GP, GI, GR, PH, and green leaf area were found to be important considerations in the alkali resistance evaluation system. Furthermore, compared with alkali-sensitive genotypes, alkali-tolerant genotypes had higher antioxidant enzyme activity, soluble osmolyte content, and lower malondialdehyde content and electrolyte leakage rate. This study provides a comprehensive and reliable method for evaluating alkali tolerance and will contribute to crop restoration by BM in alkalized ecosystems.

The present study was carried out using a hydroponics system and focused on the alkali tolerance and physiological responses of BM at the germination and seedling stages. The present study can be used as a reference for the evaluation of the alkali tolerance and could facilitate the selection of alkali-tolerant genotypes; however, there were limitations with regard to the monitoring of alkali tolerance during the entire BM growth period. Therefore, we believe that further research on the alkali resistance of BM should focus on the entire growth period in the field. In addition, the genetic diversity analysis, molecular markers identification, and genome-wide association analysis based on these 296 genotypes should be rolled out in future research studies on alkali tolerance of BM. These are what our team is doing.

## Data Availability Statement

The original contributions presented in the study are included in the article/Supplementary Material, further inquiries can be directed to the corresponding author/s.

## Author Contributions

QM and BF conceptualized and designed the study. QM, CW, and SL collected the data. QM wrote the manuscript. YY, CL, JL, and BF reviewed the manuscript. All authors have read and approved the final manuscript.

## Conflict of Interest

The authors declare that the research was conducted in the absence of any commercial or financial relationships that could be construed as a potential conflict of interest.

## Publisher's Note

All claims expressed in this article are solely those of the authors and do not necessarily represent those of their affiliated organizations, or those of the publisher, the editors and the reviewers. Any product that may be evaluated in this article, or claim that may be made by its manufacturer, is not guaranteed or endorsed by the publisher.
