# Supplementary material for: The Alkali Tolerance of Broomcorn Millet (Panicum miliaceum L.) at the Germination and Seedling Stage: The Case of 296 Broomcorn Millet Genotypes
Source: Front Plant Sci. 2021 Aug 23;12:711429. doi: 10.3389/fpls.2021.711429 (PMC8419447; doi:10.3389/fpls.2021.711429)
Supplement: Supplementary file 1 [file Table_1.docx]

**Supplementary table 1 The information of all 296 germplasm sources**

| **Code** | **Classification** | **Germplasm** | **Source** | **Code** | **Classification** | **Germplasm** | **Source** |
| --- | --- | --- | --- | --- | --- | --- | --- |
| 1 | Landrace | Huangmizi | Heilongjiang, China | 149 | Landrace | Qingyangesiniu | Gansu, China |
| 2 | Landrace | Baimizi | Heilongjiang, China | 150 | Landrace | Zhangchuanmamizi | Gansu, China |
| 3 | Landrace | Honganchunwei | Heilongjiang, China | 151 | Landrace | Heijizi | Shanxi, China |
| 4 | Landrace | Anchunwei | Heilongjiang, China | 152 | Landrace | Saigaidesi | Shanxi, China |
| 5 | Landrace | Maimizi | Heilongjiang, China | 153 | Landrace | Huimizi | Shaanxi, China |
| 6 | Landrace | Xiaomaimizi | Heilongjiang, China | 154 | Landrace | Hongyingmi | Shaanxi, China |
| 7 | Landrace | Heimizi | Heilongjiang, China | 155 | Landrace | Huimizi | Shaanxi, China |
| 8 | Landrace | 5-Feb | Heilongjiang, China | 156 | Landrace | Hangmizi | Shaanxi, China |
| 9 | Landrace | Jan-55 | Heilongjiang, China | 157 | Landrace | Heimizi | Shaanxi, China |
| 10 | Landrace | Nenshu23 | Heilongjiang, China | 158 | Landrace | Xiaotoumi | Shaanxi, China |
| 11 | Landrace | Shugu | Heilongjiang, China | 159 | Landrace | Dahuangmi | Qinghai, China |
| 12 | Landrace | 15 | Heilongjiang, China | 160 | Landrace | Huangmizi | Qinghai, China |
| 13 | Landrace | 6 | Heilongjiang, China | 161 | Landrace | Heizi | Qinghai, China |
| 14 | Landrace | 2048 | Heilongjiang, China | 162 | Landrace | Fuyubaimizi | Jilin，China |
| 15 | Landrace | 2096 | Heilongjiang, China | 163 | Landrace | Hanzhanghuangmizi | Jilin，China |
| 16 | Landrace | 2275 | Heilongjiang, China | 164 | Landrace | Qiangouhuangmizi | Jilin，China |
| 17 | Landrace | 2228 | Heilongjiang, China | 165 | Landrace | Heishu | Inner Mongolia, China |
| 18 | Landrace | Limizi | Jilin, China | 166 | Landrace | 8403/7/2 | Inner Mongolia, China |
| 19 | Landrace | Huangmizi | Jilin, China | 167 | Landrace | 8311/4/5 | Inner Mongolia, China |
| 20 | Landrace | Heimizi | Jilin, China | 168 | Landrace | Xiaohongshu | Inner Mongolia, China |
| 21 | Landrace | Heimizi | Jilin, China | 169 | Landrace | Xiaohongshu | Hebei, China |
| 22 | Landrace | Huangmizi | Liaoning, China | 170 | Landrace | Shuzi | Hebei, China |
| 23 | Landrace | Dahongshu | Liaoning, China | 171 | Landrace | Mazhayan | Hebei, China |
| 24 | Landrace | Dabaishu | Liaoning, China | 172 | Landrace | Dahuangshu | Hebei, China |
| 25 | Landrace | Mazhayan | Liaoning, China | 173 | Landrace | Dazigan | Hebei, China |
| 26 | Landrace | Jinxianhuangmizi | Liaoning, China | 174 | Landrace | Hejianbaishuzi | Hebei, China |
| 27 | Landrace | Huangqimizi | Inner Mongolia, China | 175 | Landrace | Xiaohongshu | Hebei, China |
| 28 | Landrace | Humengheinianmi | Inner Mongolia, China | 176 | Landrace | Shuzi | Hebei, China |
| 29 | Landrace | Balinzuogetashu | Inner Mongolia, China | 177 | Landrace | Qinglonghuangshuzi | Hebei, China |
| 30 | Landrace | Wuyuanheishuzi | Inner Mongolia, China | 178 | Landrace | Zijibai | Hebei, China |
| 31 | Landrace | Linheshuanglishu | Inner Mongolia, China | 179 | Landrace | Ukraine shu | Inner Mongolia, China |
| 32 | Landrace | Hanghouxiaoqingshu | Inner Mongolia, China | 180 | Landrace | Huangshu | Inner Mongolia, China |
| 33 | Landrace | Bamenghuangshuzi | Inner Mongolia, China | 181 | Landrace | Neishuyidianhong | Inner Mongolia, China |
| 34 | Landrace | Zhunqijianghuangshu | Inner Mongolia, China | 182 | Landrace | Taiyuan1036 | Heilongjiang, China |
| 35 | Landrace | Yimengliangshu 56-2 | Inner Mongolia, China | 183 | Landrace | Yu3-39 | Shanxi, China |
| 36 | Landrace | Kailubanhuangshu | Inner Mongolia, China | 184 | Landrace | Yanpibao | Jilin, China |
| 37 | Landrace | Nongwuqingshu 4 | Inner Mongolia, China | 185 | Landrace | Huangmi(shu) | Shandong, China |
| 38 | Landrace | Fengzhendabaishu | Inner Mongolia, China | 186 | Landrace | Shuzi | Shandong, China |
| 39 | Landrace | Helinhongmizi | Inner Mongolia, China | 187 | Landrace | Laoshupishuzi | Shanxi, China |
| 40 | Landrace | Wuyuanxiaohuangmi | Inner Mongolia, China | 188 | Landrace | Laolaihei | Shandong, China |
| 41 | Landrace | Bamengbaimizi | Inner Mongolia, China | 189 | Landrace | Huangshuzi | Shandong, China |
| 42 | Landrace | Bamengheimizi | Inner Mongolia, China | 190 | Landrace | Nuoshu | Hainan, China |
| 43 | Landrace | Daqidahuangmizi | Inner Mongolia, China | 191 | Landrace | Zhadashu | Tibet, China |
| 44 | Landrace | Daqiqingmizi | Inner Mongolia, China | 192 | Landrace | Gulangbangehong | Gansu, China |
| 45 | Landrace | Zhunqiziganhongmi | Inner Mongolia, China | 193 | Landrace | Dianxingziganyemi | Inner Mongolia, China |
| 46 | Landrace | Yixuandahongmi | Inner Mongolia, China | 194 | Landrace | Yemizi | Xinjiang, China |
| 47 | Landrace | Yimengshu75066-5-2 | Inner Mongolia, China | 195 | Landrace | Haiyuanziganhong | Ningxia, China |
| 48 | Landrace | Huinonghuangnianshu | Ningxia,China | 196 | Landrace | Ningxiahuangmi | Ningxia, China |
| 49 | Landrace | Gaolanyadanqing | Gansu, China | 197 | Landrace | Yangyanjingqingmizi | Ningxia, China |
| 50 | Landrace | Linghehongnianshu | Gansu, China | 198 | Landrace | Fengshuang-4 | Ningxia, China |
| 51 | Landrace | Qingshuinianmizi | Gansu, China | 199 | Landrace | Honghuamizi | Ningxia, China |
| 52 | Landrace | Xiaoshuzi | Hebei, China | 200 | Landrace | Shumi(mi) | Hebei, China |
| 53 | Landrace | Gudoubai | Hebei, China | 201 | Landrace | Baishuzi | Hebei, China |
| 54 | Landrace | Heimizi | Hebei, China | 202 | Landrace | Baishuzi | Hebei, China |
| 55 | Landrace | Nianmizi | Hebei, China | 203 | Landrace | Huangmizi | Liaoning, China |
| 56 | Landrace | Gaoliangshu | Hebei, China | 204 | Landrace | Taiyuan 3164 | Shanxi, China |
| 57 | Landrace | Xiaobaishu | Shanxi, China | 205 | Landrace | Taiyuan 3048 | Shanxi, China |
| 58 | Landrace | Liushitianxiaohongshu | Shanxi, China | 206 | Landrace | Helandahong | Ningxia, China |
| 59 | Landrace | Laolaihong | Shanxi, China | 207 | Landrace | Gugutoumi | Gansu, China |
| 60 | Landrace | Tiaozaoshu | Shanxi, China | 208 | Landrace | Tulufanmi | Xinjiang, China |
| 61 | Landrace | Wuzuishu | Shanxi, China | 209 | Landrace | Yanbeitianmi | Shanxi, China |
| 62 | Landrace | Xiaobaishu | Shanxi, China | 210 | Landrace | 78 | Former Soviet Union |
| 63 | Landrace | Dawahui | Shanxi, China | 211 | Landrace | Zhiduoaosizhi | Former Soviet Union |
| 64 | Landrace | Jiguanshu | Shanxi, China | 212 | Landrace | Sechaertuo | Poland |
| 65 | Landrace | Zaoheibai | Shanxi, China | 213 | Landrace | Huimi | Poland |
| 66 | Landrace | Huangluoshu | Shanxi, China | 214 | Landrace | 790035 | India |
| 67 | Landrace | Xiaobainianmizi | Shanxi, China | 215 | Landrace | 790051 | India |
| 68 | Landrace | Xiaoheishu | Shanxi, China | 216 | Landrace | Lahuangmi | Qinghai, China |
| 69 | Landrace | Tiaozhouruanshu | Shanxi, China | 217 | Landrace | Jinmizi | Qinghai, China |
| 70 | Landrace | Xiaoheishu | Shanxi, China | 218 | Landrace | Tuhuangmi | Qinghai, China |
| 71 | Landrace | Gouweidan | Shanxi, China | 219 | Landrace | Niuweihuang | Qinghai, China |
| 72 | Landrace | Ruanmizi | Shanxi, China | 220 | Landrace | Huimizi | Qinghai, China |
| 73 | Landrace | Baishu | Shanxi, China | 221 | Landrace | Baigetami | Qinghai, China |
| 74 | Landrace | Ruanshu | Shanxi, China | 222 | Landrace | Huanglimi | Qinghai, China |
| 75 | Landrace | Heihuiruanshu | Shanxi, China | 223 | Landrace | Erbaimi | Qinghai, China |
| 76 | Landrace | Heiruanshu | Shanxi, China | 224 | Landrace | Huangpimi | Qinghai, China |
| 77 | Landrace | Hongmizi | Shanxi, China | 225 | Landrace | A75-2 | Liaoning, China |
| 78 | Landrace | Baishu | Shanxi, China | 226 | Landrace | B75-5 | Shaanxi, China |
| 79 | Landrace | Heishu | Shanxi, China | 227 | Landrace | B75-8 | Shaanxi, China |
| 80 | Landrace | Hongruanshu | Shanxi, China | 228 | Landrace | E75-11 | Shaanxi, China |
| 81 | Landrace | Bairuanshu | Shanxi, China | 229 | Landrace | Jilinshu | Jilin, China |
| 82 | Landrace | Bendimizi | Shanxi, China | 230 | Landrace | Waiyinshu4 | The United States |
| 83 | Landrace | Xiaoqingmi | Shanxi, China | 231 | Landrace | Waiyinshu8 | The United States |
| 84 | Landrace | Huami | Shanxi, China | 232 | Landrace | A85-6 | Shanxi, China |
| 85 | Landrace | Xiaohuangshu | Shanxi, China | 233 | Landrace | A85-10 | Shanxi, China |
| 86 | Landrace | Ziganshu | Shanxi, China | 234 | Landrace | A85-29 | CAAS, China |
| 87 | Landrace | Shuzi | Shanxi, China | 235 | Landrace | A85-38 | Shaanxi, China |
| 88 | Landrace | Huangyingshu | Shanxi, China | 236 | Landrace | A85-41 | Inner Mongolia, China |
| 89 | Landrace | Baimizi | Shanxi, China | 237 | Landrace | A85-45 | Shandong, China |
| 90 | Landrace | Huangyingshu | Shanxi, China | 238 | Landrace | B85-10 | Shaanxi, China |
| 91 | Landrace | Dangdimi | Shanxi, China | 239 | Landrace | B85-20 | Shaanxi, China |
| 92 | Landrace | Huiruanmi | Shaanxi, China | 240 | Landrace | B85-25 | Shaanxi, China |
| 93 | Landrace | Ziganhongshu | Shaanxi, China | 241 | Landrace | B85-68 | Shaanxi, China |
| 94 | Landrace | Yidianhuangshu | Shaanxi, China | 242 | Landrace | A75-45 | Gansu, China |
| 95 | Landrace | Dahongmi | Shaanxi, China | 243 | Landrace | A75-70 | Shanxi, China |
| 96 | Landrace | Bairuanmi | Shaanxi, China | 244 | Landrace | E75-30 | Gansu, China |
| 97 | Landrace | Heiruanmi | Shaanxi, China | 245 | Landrace | A85-70 | Gansu, China |
| 98 | Landrace | Xiaohongruanmi | Shaanxi, China | 246 | Landrace | A85-75 | Gansu, China |
| 99 | Landrace | Huangruanmi | Shaanxi, China | 247 | Landrace | A85-80 | Shaanxi, China |
| 100 | Landrace | Saozhouruanmi | Shaanxi, China | 248 | Landrace | A85-88 | Ningxia, China |
| 101 | Landrace | Hongmi | Shaanxi, China | 249 | Landrace | A85-101 | Shaanxi, China |
| 102 | Landrace | Huangshuzi | Shaanxi, China | 250 | Landrace | B85-62 | Shaanxi, China |
| 103 | Landrace | Hongmizi | Ningxia, China | 251 | Landrace | B85-72 | Shaanxi, China |
| 104 | Landrace | Helanerhuang | Ningxia, China | 252 | Landrace | B85-90 | Shaanxi, China |
| 105 | Landrace | Misuihong | Ningxia, China | 253 | Landrace | Ziganmi | Shanxi, China |
| 106 | Landrace | Xiaohuangmizi | Ningxia, China | 254 | Breeding line | Yanshu 7 | Jilin, China |
| 107 | Landrace | Dahongmizi | Ningxia, China | 255 | Breeding line | Nianfeng 5 | Jilin, China |
| 108 | Landrace | Xijixiaohuangmi | Ningxia, China | 256 | Breeding line | Nianfeng 7 | Jilin, China |
| 109 | Landrace | Ningmi6 | Ningxia, China | 257 | Breeding line | Yimi 5 | Jilin, China |
| 110 | Landrace | Zhangyelaohuangmi | Gansu, China | 258 | Breeding line | Yumi 2 | Jilin, China |
| 111 | Landrace | Minlehongmizi | Gansu, China | 259 | Breeding line | Yumi 3 | Jilin, China |
| 112 | Landrace | Jingtaigedahong | Gansu, China | 260 | Breeding line | Longshu 21 | Jilin, China |
| 113 | Landrace | Yongdengxiaoheimi | Gansu, China | 261 | Breeding line | Longshu 23 | Jilin, China |
| 114 | Landrace | Gaolanbanlianhong | Gansu, China | 262 | Breeding line | Chishu 1 | Jilin, China |
| 115 | Landrace | Jingyuanziganhong | Gansu, China | 263 | Breeding line | Jinshu 1 | Jilin, China |
| 116 | Landrace | 60-day ziganhongmi | Gansu, China | 264 | Breeding line | Jinshu 2 | Jilin, China |
| 117 | Landrace | Huachihuangcaohongmi | Gansu, China | 265 | Breeding line | Jinshu 3 | Jilin, China |
| 118 | Landrace | Ningxianzhuyeqinghuangyingmi | Gansu, China | 266 | Breeding line | Longshu 10 | Jilin, China |
| 119 | Landrace | Ningxiandahuangnianmizi | Gansu, China | 267 | Breeding line | Jinshu 4 | Jilin, China |
| 120 | Landrace | Dongxiangduomami | Gansu, China | 268 | Breeding line | Jinshu 6 | Jilin, China |
| 121 | Landrace | Guanghehuangmi | Gansu, China | 269 | Breeding line | Jinshu 9 | Jilin, China |
| 122 | Landrace | Huangmizi | Xinjiang, China | 270 | Breeding line | Panlonghuangmi | Jilin, China |
| 123 | Landrace | Hongmi | Xinjiang, China | 271 | Breeding line | Ji 2 | Jilin, China |
| 124 | Landrace | Huangmi | Xinjiang, China | 272 | Breeding line | Ji 3 | Jilin, China |
| 125 | Landrace | Mi | Xinjiang, China | 273 | Breeding line | Ji 4 | Jilin, China |
| 126 | Landrace | Baimizi | Xinjiang, China | 274 | Breeding line | Longshu 12 | Jilin, China |
| 127 | Landrace | Heimizi | Shaanxi, China | 275 | Breeding line | Longmi 2 | Jilin, China |
| 128 | Landrace | Xiaohongmi | Shaanxi, China | 276 | Breeding line | Longmi 3 | Jilin, China |
| 129 | Landrace | Erhuangmi | Shaanxi, China | 277 | Breeding line | Longmi 4 | Jilin, China |
| 130 | Landrace | Xiaohongmi | Shaanxi, China | 278 | Breeding line | Longmi 7 | Jilin, China |
| 131 | Landrace | Jinshoushu | Unknow | 279 | Breeding line | 71049 | Jilin, China |
| 132 | Landrace | Huangjizi | Anhui, China | 280 | Breeding line | Longmi 9 | Jilin, China |
| 133 | Landrace | Baijizi | Jiangsu, China | 281 | Breeding line | Ningmi 8 | Jilin, China |
| 134 | Landrace | Xibeitianmizi(shu) | Jilin, China | 282 | Breeding line | Ningmi 9 | Jilin, China |
| 135 | Landrace | Heimizi(shu) | Jilin, China | 283 | Breeding line | Ningmi 10 | Jilin, China |
| 136 | Landrace | Xiaobaishu | Inner Mongolia, China | 284 | Breeding line | Ningmi 12 | Jilin, China |
| 137 | Landrace | Xiaohongshu | Inner Mongolia, China | 285 | Breeding line | 69-422 | Jilin, China |
| 138 | Landrace | Zhengninghongnianmi(shu) | Gansu, China | 286 | Breeding line | Ningmi 15 | Jilin, China |
| 139 | Landrace | Bailishu | Shandong, China | 287 | Breeding line | Ningmi 16 | Jilin, China |
| 140 | Landrace | Mazhayan | Shandong, China | 288 | Breeding line | Ningmi 17 | Jilin, China |
| 141 | Landrace | Baishuzi | Shandong, China | 289 | Breeding line | Liaomi 3 | Jilin, China |
| 142 | Landrace | Shuzi | Shandong, China | 290 | Breeding line | Liaomi 56 | Jilin, China |
| 143 | Landrace | Hongruanmi(shu) | Shaanxi, China | 291 | Breeding line | Gumi 21 | Jilin, China |
| 144 | Landrace | Baikemi(shu) | Shaanxi, China | 292 | Breeding line | Neimi 3 | Jilin, China |
| 145 | Landrace | Hongmi(shu) | Shaanxi, China | 293 | Breeding line | Pinmi 1 | Jilin, China |
| 146 | Landrace | 034-2 | Heilongjiang, China | 294 | Breeding line | Heitoue | Jilin, China |
| 147 | Landrace | Hongmizi | Inner Mongolia, China | 295 | Breeding line | 4452 | Jilin, China |
| 148 | Landrace | Langshan 462 | Inner Mongolia, China | 296 | Breeding line | Pinmi 2 | Jilin, China |

**Supplementary table 2 Coefficient of tolerance of millet growth characteristics to alkali stress at germination stage**

|  | Traits | Variation range (%) | Average (%) | Coefficient of variance (%) |  |
| --- | --- | --- | --- | --- | --- |
|  | RGP | 0.00-71.88 | 21.48 | 61.30 |  |
|  | RGI | 1.05-71.88 | 25.87 | 53.80 |  |
|  | RGR | 1.05-74.68 | 27.27 | 55.06 |  |
|  | RRL | 0.00-72.88 | 7.25 | 94.33 |  |
|  | RSL | 0.00-61.86 | 16.40 | 50.06 |  |
|  | RRW | 0.00-76.72 | 4.37 | 155.87 |  |
|  | RSW | 0.00-33.66 | 10.85 | 60.86 |  |
|  | RVI | 0.00-24.99 | 1.27 | 183.97 |  |
|  | RAD | 25.32-98.95 | 72.84 | 20.63 |  |

RGP: relative germination potential; RGI: relative germination index; RGR: relative germination rate; RRL: relative root length at germination; RRW: relative root fresh weight at germination; RSL: relative sprout length; RSW: relative fresh sprout weight; RVI: Relative vigor index; RAD: relative alkali damage rate.

**Supplementary table 3 Eigenvalue and contribution of each comprehensive index, loading matrix of each component, and system matrix of composition scoring (germination)**

|  | Items | Traits | Principal component | | | | | |  |
| --- | --- | --- | --- | --- | --- | --- | --- | --- | --- |
|  |  |  | Ⅰ | | Ⅱ | | Ⅲ | |  |
|  | Eigen value | | 4.125 | | 1.625 | | 1.473 | |  |
|  | Contribution (%) | | 45.828 | | 18.052 | | 16.37 | |  |
|  | Cumulative contribution (%) | | 45.828 | | 63.88 | | 80.25 | |  |
|  |  |  | Loading matrix of | System matrix of | Loading matrix of | System matrix of | Loading matrix of | System matrix of |  |
|  |  |  | each component | composition scoring | each component | composition scoring | each component | composition scoring |  |
|  |  | RGI | 0.969 | 0.206282 | 0.072 | 0.052249 | -0.213 | -0.09651 |  |
|  |  | RGR | 0.951 | 0.235033 | 0.066 | 0.044613 | -0.225 | -0.1448 |  |
|  |  | RAD | -0.951 | 0.230761 | -0.065 | 0.040717 | 0.224 | -0.15282 |  |
|  |  | RGP | 0.851 | 0.064546 | 0.085 | 0.09551 | -0.142 | 0.335661 |  |
|  |  | RRW | 0.32 | 0.03224 | -0.696 | 0.351786 | 0.561 | 0.460231 |  |
|  |  | RSW | 0.316 | 0.077493 | 0.635 | -0.42838 | 0.3557 | 0.380449 |  |
|  |  | RVI | 0.6 | 0.076709 | -0.604 | 0.390829 | 0.402 | 0.24143 |  |
|  |  | RSL | 0.133 | 0.145493 | 0.572 | -0.37205 | 0.678 | 0.272997 |  |
|  |  | RRL | 0.266 | -0.23053 | 0.155 | -0.03982 | 0.495 | 0.1519 |  |

Abbreviations are the same as in Table 1.

**Supplementary table 4 Coefficient of tolerance of millet growth characteristics to alkali stress at seedling stage**

|  | Traits | Variation range (%) | Average (%) | SD | CV(%) |  |
| --- | --- | --- | --- | --- | --- | --- |
|  | RPH | 40.97 ̶ 96.56 | 65.62 | 0.12 | 18.52 |  |
|  | RTS | 39.18 ̶ 88.78 | 67.57 | 0.11 | 16.84 |  |
|  | RGLA | 0 ̶ 87.97 | 29.46 | 0.24 | 80.91 |  |
|  | RRLs | 51.65 ̶ 142.19 | 95.00 | 0.19 | 20.15 |  |
|  | RWsl | 1.71 ̶ 79.11 | 35.02 | 0.17 | 49.66 |  |
|  | RRWs | 14.04 ̶ 83.69 | 41.51 | 0.16 | 39.51 |  |
|  | RTRL | 8.38 ̶ 80.02 | 49.78 | 0.18 | 36.36 |  |
|  | RRSA | 2.05 ̶ 86.68 | 40.60 | 0.21 | 52.71 |  |
|  | RRV | 0.24 ̶ 78.36 | 32.04 | 0.25 | 77.28 |  |

RPH: relative plant height; RTS: relative stem thickness; RGLA: relative green leaf area; RRLs: relative root length at seedling; RWsl: relative stem and leaf fresh weight; RRWs: relative root fresh weight; RTRL: relative total root length; RRSA: relative root surface area; RRV: relative root volume.

**Supplementary table 5 Eigenvalue and contribution of each comprehensive index, loading matrix of each component, and system matrix of composition scoring (germination and seedling)**

|  | Items | Traits | Principal component | | | | | | | | |  | |
| --- | --- | --- | --- | --- | --- | --- | --- | --- | --- | --- | --- | --- | --- |
|  |  | | Ⅰ | | Ⅱ | | Ⅲ | | Ⅳ | | |  | |
|  | Eigen value | | 7.467 | | 2.885 | | 1.241 | | 1.058 | | |  | |
|  | Contribution (%) | | 49.78 | | 19.231 | | 8.276 | | 7.054 | | |  | |
|  | Cumulative contribution (%) | | 49.78 | | 69.011 | | 77.284 | | 84.341 | | |  | |
|  |  |  | Loading matrix of | System matrix of | Loading matrix of | System matrix of | Loading matrix of | System matrix of | Loading matrix of | System matrix of |  | |  |
|  |  |  | each component | composition scoring | each component | composition scoring | each component | composition scoring | each component | composition scoring |  | |  |
|  |  | RPH | 0.83864119 | 0.11231336 | 0.24071487 | 0.08344722 | 0.15053478 | 0.12126524 | -0.05212223 | -0.0492574 |  | |  |
|  |  | RTS | 0.81697475 | 0.10941172 | 0.08531546 | 0.02957581 | 0.20103568 | 0.16194688 | -0.07687741 | -0.07265195 |  | |  |
|  |  | RGLA | 0.85223306 | 0.11413362 | 0.43692102 | 0.15146486 | 0.14283373 | 0.11506156 | -0.02143881 | -0.02026046 |  | |  |
|  |  | RRLs | 0.86411611 | 0.11572504 | 0.3322433 | 0.11517685 | 0.21052057 | 0.16958756 | -0.05748519 | -0.0543256 |  | |  |
|  |  | RWsl | 0.85551583 | 0.11457326 | 0.43521936 | 0.15087496 | 0.16498762 | 0.13290791 | -0.05658426 | -0.05347418 |  | |  |
|  |  | RRWs | 0.69755518 | 0.09341869 | 0.54132422 | 0.18765771 | 0.2406306 | 0.19384309 | -0.10417845 | -0.09845242 |  | |  |
|  |  | RTRL | 0.63626226 | 0.08521016 | 0.22280328 | 0.07723792 | -0.43625139 | -0.35142793 | 0.28280329 | 0.26725938 |  | |  |
|  |  | RRSA | 0.67734915 | 0.09071264 | 0.21149135 | 0.07331647 | -0.49248287 | -0.39672593 | 0.24590831 | 0.23239229 |  | |  |
|  |  | RRV | 0.61263154 | 0.08204546 | 0.09234122 | 0.03201139 | -0.58999542 | -0.47527842 | 0.18556646 | 0.17536704 |  | |  |
|  |  | RGP | 0.69767961 | 0.09343536 | -0.5567794 | -0.19301547 | 0.00515982 | 0.00415656 | -0.12150572 | -0.11482732 |  | |  |
|  |  | RGI | 0.71945183 | 0.09635116 | -0.65861495 | -0.22831821 | -0.05739793 | -0.04623764 | -0.19487247 | -0.18416156 |  | |  |
|  |  | RGR | 0.69833078 | 0.09352256 | -0.6572371 | -0.22784055 | -0.06919154 | -0.05573814 | -0.21365788 | -0.20191446 |  | |  |
|  |  | RRL | 0.31905614 | 0.04272896 | -0.35772196 | -0.12400939 | 0.42153952 | 0.33957659 | 0.55989785 | 0.52912381 |  | |  |
|  |  | RSL | 0.28870735 | 0.03866456 | -0.44628354 | -0.15471052 | 0.23872993 | 0.19231198 | 0.63096906 | 0.59628869 |  | |  |
|  |  | RAD | -0.69899204 | -0.09361112 | 0.65567677 | 0.22729965 | 0.06857218 | 0.0552392 | 0.21641336 | 0.20451849 |  | |  |

RPH: relative plant height; RTS: relative stem thickness; RGLA: relative green leaf area; RRLs: relative root length at seedling; RWsl: relative stem and leaf fresh weight; RRWs: relative root fresh weight; RTRL: relative total root length; RRSA: relative root surface area; RRV: relative root volume; RGP: relative germination potential; RGI: relative germination index; RGR: relative germination rate; RRL: relative root length at germination; RSL: relative sprout length; RAD: relative alkali damage rate.

**Supplementary table 6 Eigenvalue and contribution of each comprehensive index, loading matrix of each component, and system matrix of composition scoring (seedling)**

| Items | Traits | Principal component | |  |  |
| --- | --- | --- | --- | --- | --- |
|  |  | Ⅰ |  | Ⅱ |  |
| Eigen value |  | 6.103 |  | 1.213 |  |
| Contribution (%) | | 67.812 |  | 13.479 |  |
| Cumulative contribution (%) | | 67.812 |  | 81.291 |  |
|  |  | Loading matrix of | System matrix of | Loading matrix of | System matrix of |
|  |  | each component | composition scoring | each component | composition scoring |
|  | RPH | 0.87003362 | 0.14255588 | -0.14970405 | -0.12340333 |
|  | RTS | 0.7976589 | 0.13069721 | -0.17617608 | -0.14522463 |
|  | RGLA | 0.94928256 | 0.1555409 | -0.16833388 | -0.13876019 |
|  | RRLs | 0.92236737 | 0.15113082 | -0.22717186 | -0.18726123 |
|  | RWsl | 0.95040983 | 0.15572561 | -0.20676342 | -0.17043825 |
|  | RRWs | 0.83771316 | 0.13726014 | -0.32360187 | -0.26674996 |
|  | RTRL | 0.67979803 | 0.11138559 | 0.48212211 | 0.39742061 |
|  | RRSA | 0.71544996 | 0.1172272 | 0.54154256 | 0.4464018 |
|  | RRV | 0.61559098 | 0.1008652 | 0.6376175 | 0.52559783 |

RPH: relative plant height; RTS: relative stem thickness; RGLA: relative green leaf area; RRLs: relative root length at seedling; RWsl: relative stem and leaf fresh weight; RRWs: relative root fresh weight; RTRL: relative total root length; RRSA: relative root surface area; RRV: relative root volume.

**Supplementary table 7 The alkali tolerance (F-Value) of 111 germplasm sources (germination and germination)**

| **Code** | **FS** | **FGS** | **Code** | **FS** | **FGS** | **Code** | **FS** | **FGS** | **Code** | **FS** | **FGS** |
| --- | --- | --- | --- | --- | --- | --- | --- | --- | --- | --- | --- |
| 4 | 42.32046 | 38.79614 | 85 | 38.44364 | 34.40814 | 168 | 43.03312 | 39.17925 | 224 | 49.09432 | 39.80618 |
| 7 | 38.44364 | 35.75825 | 89 | 54.8266 | 42.01118 | 171 | 22.36251 | 22.80943 | 226 | 22.36251 | 22.75922 |
| 11 | 56.98552 | 43.53061 | 94 | 53.35922 | 41.48111 | 172 | 32.59459 | 29.17052 | 219 | 27.70848 | 24.23178 |
| 12 | 56.1602 | 42.53239 | 97 | 14.67451 | 15.9115 | 27 | 37.04517 | 24.70419 | 232 | 42.96772 | 36.31856 |
| 17 | 57.39944 | 43.59086 | 101 | 54.95778 | 40.02358 | 175 | 41.6194 | 25.89377 | 238 | 34.09735 | 28.58932 |
| 22 | 47.1274 | 37.69901 | 106 | 44.54475 | 40.3366 | 176 | 23.23993 | 20.99542 | 244 | 46.05704 | 35.67919 |
| 29 | 37.04517 | 24.84701 | 114 | 50.10672 | 36.24364 | 178 | 32.78577 | 19.96203 | 245 | 25.71984 | 24.80457 |
| 32 | 37.04517 | 23.20225 | 115 | 55.57121 | 41.35048 | 180 | 36.08418 | 25.6844 | 246 | 35.47906 | 25.05465 |
| 33 | 53.6403 | 41.35737 | 117 | 73.24595 | 54.66399 | 182 | 27.70848 | 23.26327 | 247 | 44.48881 | 31.64237 |
| 36 | 53.43273 | 37.84783 | 121 | 42.81745 | 37.50059 | 184 | 28.6551 | 21.55802 | 248 | 41.90608 | 31.35256 |
| 37 | 45.04673 | 32.39331 | 123 | 22.79926 | 20.9817 | 186 | 37.97177 | 32.0743 | 256 | 29.69597 | 23.51602 |
| 39 | 45.04673 | 33.6621 | 125 | 53.16187 | 41.65419 | 190 | 42.45961 | 34.75774 | 258 | 66.48168 | 46.48477 |
| 40 | 45.49739 | 40.46461 | 126 | 22.79926 | 21.67287 | 191 | 46.42571 | 35.24812 | 260 | 37.93197 | 27.63559 |
| 42 | 43.026 | 40.50555 | 127 | 22.36251 | 20.85306 | 193 | 25.71984 | 24.58045 | 261 | 44.64439 | 33.91462 |
| 43 | 66.01344 | 46.71103 | 130 | 52.65984 | 39.78011 | 195 | 45.55403 | 31.99541 | 265 | 53.31164 | 37.46505 |
| 44 | 57.8837 | 42.96811 | 132 | 40.68355 | 36.08039 | 196 | 73.54632 | 52.30356 | 270 | 28.828 | 21.54595 |
| 46 | 55.34505 | 40.32453 | 135 | 51.02466 | 40.91709 | 197 | 54.98624 | 40.68791 | 275 | 26.2212 | 26.56619 |
| 51 | 47.71067 | 41.21021 | 138 | 54.10042 | 40.10536 | 198 | 69.14981 | 50.88762 | 279 | 24.67794 | 23.1982 |
| 52 | 73.71889 | 55.6882 | 145 | 42.75957 | 38.01954 | 204 | 46.47615 | 33.34696 | 282 | 33.97457 | 29.4016 |
| 53 | 32.14007 | 26.66034 | 147 | 53.07066 | 38.41262 | 206 | 39.09023 | 28.59888 | 285 | 70.53953 | 52.02476 |
| 56 | 42.21838 | 39.1923 | 150 | 71.63929 | 52.43047 | 210 | 41.55749 | 35.45197 | 286 | 51.62187 | 38.01816 |
| 62 | 38.44364 | 34.01935 | 151 | 72.51854 | 53.66267 | 213 | 25.71984 | 24.83912 | 287 | 49.00744 | 37.53968 |
| 64 | 50.67716 | 39.09292 | 154 | 74.0871 | 54.85243 | 215 | 41.42724 | 27.99158 | 291 | 37.42175 | 27.40457 |
| 68 | 50.60156 | 36.22982 | 159 | 26.2212 | 27.38602 | 217 | 41.4609 | 26.38222 | 292 | 44.16016 | 31.93703 |
| 74 | 14.67451 | 16.52641 | 160 | 49.41252 | 33.99349 | 218 | 78.4319 | 55.43487 | 293 | 45.79133 | 33.43586 |
| 76 | 49.71804 | 40.06246 | 163 | 46.85164 | 34.20876 | 219 | 34.90379 | 24.36919 | 294 | 28.22327 | 20.90609 |
| 80 | 35.07386 | 23.60952 | 165 | 55.67138 | 41.93213 | 220 | 41.75355 | 34.79838 | 295 | 36.46597 | 26.9674 |
| 81 | 32.14007 | 26.35337 | 166 | 26.2212 | 26.43795 | 221 | 70.48416 | 50.94091 |  |  |  |
