# Supplementary material for: The Alkali Tolerance of Broomcorn Millet (Panicum miliaceum L.) at the Germination and Seedling Stage: The Case of 296 Broomcorn Millet Genotypes
Source: Front Plant Sci. 2021 Aug 23;12:711429. doi: 10.3389/fpls.2021.711429 (PMC8419447; doi:10.3389/fpls.2021.711429)
Supplement: Supplementary file 2 [file Data_Sheet_1.docx]

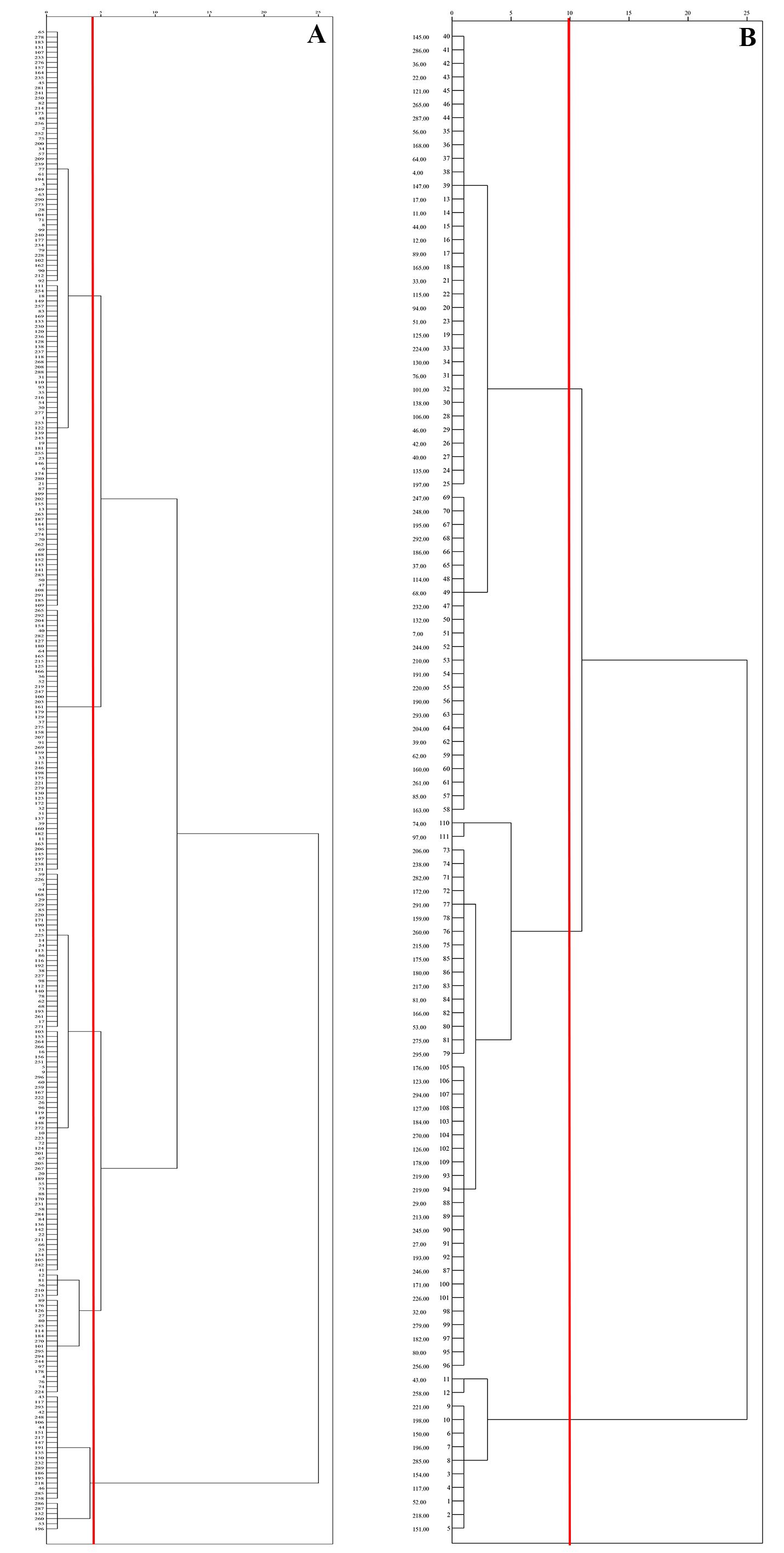


Supplementary figure 1. Alkali tolerance clustering. (A): the alkali tolerance clustering of 296 broomcorn millet germplasm resources at the germination stage; (B): the comprehensive alkali tolerance clustering of 111 broomcorn millet germplasm resources combined with the germination and seedling stages.
